# Supplementary material for: Changes in the length of speeches in the plays of William Shakespeare and his contemporaries: A mixed models approach
Source: PLoS One. 2023 Apr 21;18(4):e0282716. doi: 10.1371/journal.pone.0282716 (PMC10121026; doi:10.1371/journal.pone.0282716)
Supplement: S1 File — (HTML) [file pone.0282716.s001.html]

S1-Speech-length.knit


### Changes in the length of speeches in the plays of William Shakespeare and his contemporaries: a mixed models approach

Colyvas, Craig and Egan

# Supplementary 1 - Analysis

This supplementary file contains all the details behind the analysis
including computer output, tables and figures and commentary on the
analysis. This is provided for the reader interested in additional
information to that in the manuscript. The data files and R markdown
script that produced the supplementary documents are also available so
that all the analyses, tables, figures and text could be reproduced.
Most of the analysis of the mode was carried out in Supplementary 2 in
which 2 modelling distributions were compared, the normal and the
Poisson. Only the final model for the mode based on a LMM from
Supplementary 2 has been transferred and used here. In Supplementary 3
the speech length distributions for 15 plays with 2 modes (and in one
case 3) that had equal frequency were resolved so that only 1 mode was
used for the analysis here.

```
# Read the 2 sources of data that had been previously processed using the file 
# Speech length modelling V4 - create summary data sets.Rmd

# Speech length data
speeeches=read.table("Speeches.txt",header=T,sep="\t",row.names = NULL,quote = "" )
# names(speeeches)

# Order the time and proportion of verse lines factors
speeeches$f_period3=factor(speeeches$period3,levels=c("< 1597","1597-1602","> 1602"))

# Summary by play
plays=read.table("Plays.txt",header=T,sep="\t",row.names = NULL,quote = "")
#names(plays)
#nrow(plays)

# Modify the mode for 2 plays based on the analysis in S3 Speech length mode adjustment.Rmd
plays$mode[plays$TextTitle == "Edmond Ironside"] = 8
plays$mode[plays$TextTitle == "Massacre at Paris"] = 8

# Order the time and proportion of verse lines factors
plays$f_period3=factor(plays$f_period3,levels=c("< 1597","1597-1602","> 1602"))
plays$verse_grp=factor(plays$verse_grp,levels=c("< 0.30","0.30 to < 0.70","0.70 to 0.90",">0.90"))

# Remove 2 plays for the primary analysis - see supplementary 2 for the reasons for excluding these
# The Tragedy of Miriam (extreme mode 33) and Philotas (large Pearson residual, 9 SDs higher than model prediction)
plays3 = filter(plays,mode < 33 & TextTitle != "Philotas")

# As Daniel Samuel now has only 1 play left his remaining play should be assigned to the Single play Author group
plays3$Author2[59] = "Single Play Author"

# Order by date and play title for use in the cusum analysis
plays2=arrange(plays3,Date,TextTitle)

# Remove the PlayType2 = "Other" category - for additional modeling for the mean of speech length
plays_ap = filter(plays3,PlayType2 == "Adult Professional")
```

## Change of mode over time

In S1 Fig 1 below the mode is plotted over time with a small amount
of random noise added to better display overplotted values (called
jittering). A loess smoother is used to show the general trend over
time. The smoothing line suggests the mode begins to drop from a mode
with mean a little lower than 9 at 1590 and stabilises at a lower level
with mean of about 5 from 1610.

A striking feature in the plot is the appearance of quite a number of
plays with a mode of 4 from 1597 onwards with very few plays with modes
larger than 7 in the post 1600 period. The two dotted lines vertical
lines in the plot indicate what our analysis suggests is a transitional
period for this change. The cumulative sum (cusum) analysis analysis
below explains how this was obtained.

S1 Fig 1 Plot of mode for all plays with the solid line formed by a
loess smoother showing the trend to lower values over time. Two dotted
vertical lines border the period 1597 to 1602. The mode was jittered.

## Cumulative sum (cusum) chart analysis

S1 Fig 2 Upper plot is the cusum plot of mode for each play. The
approximately straight line segments with different slopes indicate the
time periods that are interpreted as steps. Time change points are where
the line segments change slope. The lower plot is the mode data with the
solid line showing the step function interpretation for the mean of the
mode using the change points in the cusum plot.

### Interpretation of the cusum plot

Essentially a cusum (cumulative sum) chart is used to interpret a
process in terms of steps and helps in identifying the time at which
step changes occurred. As previous authors had identified there was a
change over time this charting tool was used to help in more accurately
identifying when changes in the mode of plays took place. Cusum charts
are usually plotted with time data that is obtained at regular
intervals. It has been adapted here to the monitor the process of play
production which is very irregular in terms of time. This does not
matter however so long as the underlying process is operating at the
same constant level till a change occurs to a new constant level post
the change point. The irregular sampling intervals within or between
years simply serves to examine the underlying mean level within each
time step. The main purpose here being to identify if the process has
changed compared to previous time values. Multiple plays written within
a year are like random samples within that year as they are ordered
alphabetically based on play title. Therefore the best that could be
achieved by the chart with the play data would be to identify changes to
the nearest year.

The first main segment of the cusum plot, observations 1 to 80
(ending at the second vertical dotted line) had a positive slope
indicating the mode was at a level (step) above the (arbitrarily chosen)
target value of 6. As the slope of the line was generally linear this
indicated that the mode was stable. Within this first segment however
there could be 2 sections. Observations 1 to 14 (years 1538 to 1583)
appear to have a steeper slope so if this was treated as the first step
the mode would be on average 9.9. For the second section of observations
15 to 80, the slope was less steep than the first section indicating a
lower mode, mean of 8.5. In the second segment the slope had changed to
approximately horizontal indicating the mean was approximately the same
as the target level for the plot, observations 81-126 (years 1597 to
1602), with mean mode 6.1. Finally in the last segment observations
127-274 (years 1603 to 1642) the mode was at it lowest level with mean
4.7.

### List of plays around the 3 change point areas of the cusum plot

In 4 tables that follow, S1 Table 1a, 1b, 1c & 1d, is a listing
of plays either side of the change points marked in the cusum plot above
by the vertical dotted lines. The marker lines in the cusum plot
indicate the first point of a new process step. The steps identified
from the 3 change points shown in cusum plot (15, 81 and 127, each being
the first point in the new step section of the plot ) are for 4 groups
of observations 1-14, 15-80, 81-126 and 127-273. So the step change
interpretation of the process of producing plays with certain modes can
be summarised as having 4 periods with the mode progressively reducing
over time from a high of 9.9 to a sustained low of 4.6 after 1602. The
full list of plays within the transition period is also shown to allow
examination of the authors who began writing plays with modes of 4
compared to those who continued with modes of 8. After 1602 most plays
had modes of 4.

Firstly some plays from the first section before 1597, S1 Table 1a.
Initially those comprising the change point for the first section
(observations 1-14) and 6 from the change point onwards.

S1 Table 1a Plays prior to 1597, 14 plays in the first step and
then the first 6 plays in the second step


| Obs | Date | Time Period | Mode | Text title | Author |
| --- | --- | --- | --- | --- | --- |
| 1 | 1538 | < 1597 | 10 | King Johan | Bale John |
| 2 | 1552 | < 1597 | 10 | Ralph Roister Doister | Udall Nicholas |
| 3 | 1553 | < 1597 | 13 | Gammer Gurton�s Needle | Uncertain |
| 4 | 1559 | < 1597 | 10 | Patient and Meek Grissel | Phillip John |
| 5 | 1562 | < 1597 | 15 | Ferrex and Porrex | Sackville Thomas |
| 6 | 1564 | < 1597 | 9 | Appius and Virginia | Uncertain |
| 7 | 1564 | < 1597 | 10 | Damon and Pithias | Edwards Richard |
| 8 | 1567 | < 1597 | 7 | Tancred and Gismund | Wilmot et al. |
| 9 | 1570 | < 1597 | 11 | Clyomon and Clamydes | Uncertain |
| 10 | 1581 | < 1597 | 6 | Arraignment of Paris | Peele George |
| 11 | 1581 | < 1597 | 11 | Three Ladies of London | Wilson Robert |
| 12 | 1582 | < 1597 | 10 | Rare Triumphs of Love and Fortune | Uncertain |
| 13 | 1583 | < 1597 | 10 | Campaspe | Lyly John |
| 14 | 1583 | < 1597 | 6 | Sappho and Phao | Lyly John |
| 15 | 1584 | < 1597 | 4 | Fedele and Fortunia | Munday Anthony (?) |
| 16 | 1585 | < 1597 | 6 | Gallathea | Lyly John |
| 17 | 1586 | < 1597 | 8 | Dido Queen of Carthage | Marlowe and Nashe |
| 18 | 1586 | < 1597 | 9 | Famous Victories of Henry the Fifth | Uncertain |
| 19 | 1587 | < 1597 | 15 | 1 Tamburlaine the Great | Marlowe Christopher |
| 20 | 1587 | < 1597 | 8 | Alphonsus King of Aragon | Greene Robert |

Then in S1 Table 1b up to the end of the 2nd step that ends at
observation 80, steps 1 and 2 comprising the first time period.

S1 Table 1b Plays prior to 1597, 11 plays at the end of the
second step


|  | Date | Time Period | Mode | Text title | Author |
| --- | --- | --- | --- | --- | --- |
| 70 | 1594 | < 1597 | 16 | Love of David and Fair Bathsheba | Peele George |
| 71 | 1594 | < 1597 | 8 | Two Lamentable Tragedies | Yarington Robert |
| 72 | 1595 | < 1597 | 9 | Love�s Labor�s Lost | Shakespeare William |
| 73 | 1595 | < 1597 | 9 | Midsummer Night�s Dream | Shakespeare William |
| 74 | 1595 | < 1597 | 8 | Richard the Second | Shakespeare William |
| 75 | 1595 | < 1597 | 9 | Romeo and Juliet | Shakespeare William |
| 76 | 1596 | < 1597 | 9 | Blind Beggar of Alexandria | Chapman George |
| 77 | 1596 | < 1597 | 8 | Captain Thomas Stukeley | Uncertain |
| 78 | 1596 | < 1597 | 9 | King John | Shakespeare William |
| 79 | 1596 | < 1597 | 8 | Merchant of Venice | Shakespeare William |
| 80 | 1596 | < 1597 | 8 | Mustapha | Greville Fulke |

The whole of the transition period (1597-1602) is provided in S1
Table 1c.

S1 Table 1c Plays in the transition 1597-1602, the third
step


|  | Date | Time Period | Mode | Text title | Author |
| --- | --- | --- | --- | --- | --- |
| 81 | 1597 | 1597-1602 | 6 | 1 Henry the Fourth | Shakespeare William |
| 82 | 1597 | 1597-1602 | 6 | 2 Henry the Fourth | Shakespeare William |
| 83 | 1597 | 1597-1602 | 4 | Case Is Altered | Jonson Ben |
| 84 | 1597 | 1597-1602 | 8 | Edmond Ironside | Uncertain |
| 85 | 1597 | 1597-1602 | 9 | Humorous Day�s Mirth | Chapman George |
| 86 | 1597 | 1597-1602 | 4 | Merry Wives of Windsor | Shakespeare William |
| 87 | 1598 | 1597-1602 | 4 | 1 Two Angry Women of Abingdon | Porter Henry |
| 88 | 1598 | 1597-1602 | 4 | Death of Robert Earl of Huntingdon | Munday and Chettle |
| 89 | 1598 | 1597-1602 | 8 | Downfall of Robert Earl of Huntingdon | Munday and Chettle |
| 90 | 1598 | 1597-1602 | 9 | Englishmen for My Money | Haughton William |
| 91 | 1598 | 1597-1602 | 5 | Every Man in His Humor | Jonson Ben |
| 92 | 1598 | 1597-1602 | 6 | Much Ado About Nothing | Shakespeare William |
| 93 | 1598 | 1597-1602 | 8 | Virtuous Octavia | Brandon Samuel |
| 94 | 1599 | 1597-1602 | 9 | 1 Edward the Fourth | Heywood Thomas |
| 95 | 1599 | 1597-1602 | 4 | 1 Sir John Oldcastle | Wilson et al. |
| 96 | 1599 | 1597-1602 | 8 | 2 Edward the Fourth | Heywood Thomas |
| 97 | 1599 | 1597-1602 | 4 | Antonio and Mellida | Marston John |
| 98 | 1599 | 1597-1602 | 5 | As You Like It | Shakespeare William |
| 99 | 1599 | 1597-1602 | 6 | Every Man Out of His Humor | Jonson Ben |
| 100 | 1599 | 1597-1602 | 8 | Henry the Fifth | Shakespeare William |
| 101 | 1599 | 1597-1602 | 8 | Histriomastix | Marston John |
| 102 | 1599 | 1597-1602 | 4 | Julius Caesar | Shakespeare William |
| 103 | 1599 | 1597-1602 | 9 | Larum for London | Uncertain |
| 104 | 1599 | 1597-1602 | 4 | Look About You | Uncertain |
| 105 | 1599 | 1597-1602 | 9 | Old Fortunatus | Dekker Thomas |
| 106 | 1599 | 1597-1602 | 4 | Shoemaker�s Holiday | Dekker Thomas |
| 107 | 1599 | 1597-1602 | 9 | Warning for Fair Women | Uncertain |
| 108 | 1600 | 1597-1602 | 4 | Antonio�s Revenge | Marston John |
| 109 | 1600 | 1597-1602 | 4 | Cynthia�s Revels | Jonson Ben |
| 110 | 1600 | 1597-1602 | 8 | Devil and his Dame | Haughton William |
| 111 | 1600 | 1597-1602 | 8 | Jack Drum�s Entertainment | Marston John |
| 112 | 1600 | 1597-1602 | 4 | Lust�s Dominion | Uncertain |
| 113 | 1600 | 1597-1602 | 9 | Thomas Lord Cromwell | Uncertain |
| 114 | 1601 | 1597-1602 | 4 | All Fools | Chapman George |
| 115 | 1601 | 1597-1602 | 4 | Hamlet Prince of Denmark | Shakespeare William |
| 116 | 1601 | 1597-1602 | 4 | Poetaster | Jonson Ben |
| 117 | 1601 | 1597-1602 | 9 | Satiromastix | Dekker Thomas |
| 118 | 1601 | 1597-1602 | 9 | Sir Thomas More | Uncertain |
| 119 | 1601 | 1597-1602 | 4 | Twelfth Night | Shakespeare William |
| 120 | 1601 | 1597-1602 | 4 | What You Will | Marston John |
| 121 | 1602 | 1597-1602 | 9 | Gentleman Usher | Chapman George |
| 122 | 1602 | 1597-1602 | 5 | Hoffman | Chettle Henry |
| 123 | 1602 | 1597-1602 | 5 | May Day | Chapman George |
| 124 | 1602 | 1597-1602 | 8 | Sir Giles Goosecap | Chapman George |
| 125 | 1602 | 1597-1602 | 5 | Sir Thomas Wyatt | Dekker and Webster |
| 126 | 1602 | 1597-1602 | 4 | Troilus and Cressida | Shakespeare William |

This additional table of author by mode was created to examine if
some authors might have been the first in transitioning their writing to
use simpler forms of writing as indicated by the mode of the play being
about 4, 5 or 6, compared to other authors still not switching style
(with modes 8 or 9). From 1583 to 1596 the predominant modes were 8 or 9
with modes of around 4 being relatively uncommon. However from 1597 the
prevalence of plays with modes of 4 increased considerably suggesting
this was a time of transition where perhaps some authors were taking the
lead. This is the reason for printing out all the plays in this segment
- to examine which authors were writing plays with smaller modes. This
transitional pattern is most obvious in the step function plot above (S1
Fig 2, lower plot of the cusum plot pair).

```
##                      mode
## Author                4 5 6 8 9
##   Brandon Samuel      0 0 0 1 0
##   Chapman George      1 1 0 1 2
##   Chettle Henry       0 1 0 0 0
##   Dekker and Webster  0 1 0 0 0
##   Dekker Thomas       1 0 0 0 2
##   Haughton William    0 0 0 1 1
##   Heywood Thomas      0 0 0 1 1
##   Jonson Ben          3 1 1 0 0
##   Marston John        3 0 0 2 0
##   Munday and Chettle  1 0 0 1 0
##   Porter Henry        1 0 0 0 0
##   Shakespeare William 5 1 3 1 0
##   Uncertain           2 0 0 1 4
##   Wilson et al.       1 0 0 0 0
```

From this we see that a number of authors had switched to plays with
lower modes (4,5 or 6) but that there were still some plays being
produced at the longer lengths with modes 8 to 9. Among these were
Chapman, Dekker and Marston. Shakespeare had largely transitioned with
all plays having lower modes apart from Henry the Fifth with mode 8.
Jonson only had lower modes for his 5 plays. Interestingly there were no
plays with a mode of 7, reflecting perhaps the 2 distinct groupings,
lower modes and higher modes.

Finally in S1 Table 1d the first few plays after the end of the
transition period, 1603 and beyond. The predominance of modes of 4 from
1603 is quite clear in the step function plot of S1 Fig 2. Also plays
with modes higher than 6 are relatively rare. Contrast this with the pre
1597 period where the opposite was true.

S1 Table 1d First plays after the transition period, the fourth
step, > 1602


|  | Date | Time Period | Mode | Text title | Author |
| --- | --- | --- | --- | --- | --- |
| 127 | 1603 | > 1602 | 4 | All�s Well That Ends Well | Shakespeare William |
| 128 | 1603 | > 1602 | 7 | Family of Love | Middleton Thomas |
| 129 | 1603 | > 1602 | 4 | Measure for Measure | Shakespeare & Middleton |
| 130 | 1603 | > 1602 | 4 | Phoenix | Middleton Thomas |
| 131 | 1603 | > 1602 | 4 | Woman Killed with Kindness | Heywood Thomas |
| 132 | 1604 | > 1602 | 4 | 1 Fair Maid of the West | Heywood Thomas |
| 133 | 1604 | > 1602 | 9 | 1 If You Know Not Me You Know Nobody | Heywood Thomas |
| 134 | 1604 | > 1602 | 5 | 1 Jeronimo | Uncertain |
| 135 | 1604 | > 1602 | 4 | Bussy D�Ambois | Chapman George |

# Speech Length Distributions

Following are 2 tables, S1 Table 2 showing the frequency of plays
within 4 categories for the proportion of verse in a play and S1 Table 3
the frequency in the 3 time period groups. In addition in S1 Fig 3 a
histogram of the proportion of verse lines within each play with
vertical markers showing the divisions chosen to create the categorical
proportion of verse variable that was used for creating groups for
plotting of distributions.

S1 Table 2 Number of plays and percentages in the 4 groupings
based on proportion of verse in a play, N=273 plays

| Verse group | Count | % |
| --- | --- | --- |
| < 0.30 | 37 | 13.6 |
| 0.30 to < 0.70 | 56 | 20.5 |
| 0.70 to 0.90 | 76 | 27.8 |
| >0.90 | 94 | 34.4 |
| NA | 10 | 3.7 |

S1 Table 3 Number of plays and percentages in the 3 time
periods, N=273 plays

| Time Period | Count | % |
| --- | --- | --- |
| < 1597 | 80 | 29.3 |
| 1597-1602 | 46 | 16.8 |
| > 1602 | 147 | 53.8 |

S1 Fig 3 Distribution of lengths of speeches (the range being from a
mode of 1 word to a mode of 30 words) by verse and time period groups.
The vertical markers are at speech lengths 4, 8 and 16.

S1 Fig 4 Distribution of lengths of speeches (range 1 to 30) by verse
and time period groups. The vertical markers are at speech lengths 4, 8
and 16 words.

S1 Fig 3 shows the distribution of proportion of verse lines within
plays for the 4 verse groupings. In S1 Fig 4 the 12 histograms are
arranged in 4 vertical panels for the 4 verse groupings of proportion of
lines within the play and 3 horizontal panels for the time period
groups. Overall it is clear the speech length distributions depend on
both these variables. Within the first row of plots for the period
before 1597 when the proportion of verse in a play is below 0.30 the
distribution is a relatively smooth skewed shape. However in the next 3
panels with increasing proportion of verse within a play (0.30 to just
below 0.70 through to above 0.90) an obvious and dominant mode appears
in the region of speech lengths 8 to 9 words. Interestingly a second but
smaller peak becomes obvious at 16 for the two highest verse groups as
well as a third weak peak at 24. The fact that we see higher peaks as
the proportion of verse in a play increases supports the notion these
peaks are related to the number of words in a line of verse. The
relative strength of the peak at 8 or 9 suggests a predominance of
speeches of 1 line of verse, with the other two peaks at 16 and 24 being
related to 2 or 3 lines of verse.

The pattern of modes changes dramatically in the bottom row of panels
for the post-1602 period where there is only a single peak at 4 for all
3 of the higher verse groups distributions is followed by only a slight
hint of the peaks at 16 and 24 remaining. The very lowest verse group
below 0.30 which did not have a strong multi-peak distribution in the
pre-1597 graph also shows a shift of its mode from 6 in the pre-1597
period to a lower value of 4 for the post-1602 period.

The middle row of plots for the period 1587 to 1602 contains a
mixture of the distributions of the earlier and later periods. The
appearance of peaks at 4 for the 3 highest verse groups, while the other
modes at 8 to 9, 16 and 24 are still evident, indicative of the
transition between the pre-1597 and post-1602 distributions. This can be
seen in S1 Fig 2 where the mode is plotted over time. Clearly there is a
mixture of peaks in the transitional period with some plays having modes
of about 8 and quite a number having modes of 4. After 1602 the
predominance of plays with modes of 4 is apparent.

In summary the change from a the multi-peak distribution in the
period prior to 1597 to a primarily uni-peak distribution after 1602
points to, irrespective of the proportion of verses in a play, a
substantial change in the style of the writing that began about 1597 and
was complete post 1602.

## Verse proportion with time

A LMM analysis was run with verse proportion now as the outcome
variable rather than predictor to see how it varied between the time
groups. There was a significant relationship, the pre 1597 period had a
higher mode compared to the other 2 time periods. From the plot of
estimated marginal means (EMM) S1 Fig 5 (see also the emmean and
pairwise comparison tables) the mean proportion of verse in plays was
about 78% before 1597, with significantly reduced levels in the
subsequent periods, 59% in the transition period 1597-1602 and after
1602 68%. This suggests a change in writing style related to verse
proportion over time as does the speech length analysis below which is
the main subject of this investigation.

Was this change in verse proportion over time the underlying reason
for the speech length changes observed in the mode and for median and
mean (see analysis below)? This question was examined by some additional
data exploration and analysis.

S1 Fig 5 Estimated marginal means and 95% CIs of the proportion of verse
in plays for the three time periods

```
## Type III Analysis of Variance Table with Satterthwaite's method
##           Sum Sq Mean Sq NumDF  DenDF F value Pr(>F)    
## f_period3 0.7748  0.3874     2 251.56  7.4784  7e-04 ***
## ---
## Signif. codes:  
## 0 '***' 0.001 '**' 0.01 '*' 0.05 '.' 0.1 ' ' 1
```

```
##  f_period3 emmean     SE   df lower.CL upper.CL
##  < 1597     0.779 0.0528 53.2    0.673    0.885
##  1597-1602  0.586 0.0558 71.6    0.475    0.697
##  > 1602     0.670 0.0462 36.9    0.576    0.763
## 
## Degrees-of-freedom method: kenward-roger 
## Confidence level used: 0.95
```

```
##  contrast             estimate     SE  df t.ratio p.value
##  < 1597 - (1597-1602)   0.1928 0.0503 259   3.829  0.0002
##  < 1597 - > 1602        0.1089 0.0455 251   2.395  0.0174
##  (1597-1602) - > 1602  -0.0839 0.0452 255  -1.857  0.0644
## 
## Degrees-of-freedom method: kenward-roger
```

# Is there an effect on speech length due to the proportion of verse lines?

An initial data exploration was carried out to understand if there
was a relationship between the mode and proportion of verse lines. The
reason for this being that the various modes (primary,secondary and
tertiary) evident in S1 Fig 4 were thought to be the result of the verse
structure of the writing so the possibility that the mode was influenced
by the proportion of verse in a play was investigated. In the S1 Fig 6
below the proportion of verse within plays was plotted against the mode
for each of the 3 time periods. The logic behind this being if there was
a relationship between verse proportion and the mode it should have been
evident within each time period, each period showing a similar
relationship. For example if all 3 time periods had negative slopes a
possible interpretation might be as the proportion of verse in a play
increased the decrease in the mode could have been due to longer text
speeches being replaced by more text in shorter speeches using verse
with the additional effect on leading to multiple peaks/modes in the
distributions in S1 Fig 4.The lines of best fit were based on a GAM
(Generalised Additive Model) smoother. A consistent pattern was not
observed with all fitted relationships being similar. Rather there was a
diminishing slope over the 3 time periods.

S1 Fig 6 Mode of speech lengths plotted against proportion of verse in a
play. A GAM smoothing line and 95% CI was added within each time period

This was also examined statistically using a LMM for the mode
treating proportion of verse as a linear predictor and author as a
random effect. The results shown below indicate that there is a
significant divergence in the slopes (the interaction between verse
proportion and time period being significant, p=.002).

```
# Interaction model
pv3=lmer(mode~verse_prop*f_period3 + (1|Author2),data=plays3)
#summary(pv3)
anova(pv3)
```

```
## Type III Analysis of Variance Table with Satterthwaite's method
##                      Sum Sq Mean Sq NumDF  DenDF F value
## verse_prop           19.019  19.019     1 206.15  5.0155
## f_period3             4.878   2.439     2 151.34  0.6432
## verse_prop:f_period3 66.438  33.219     2 183.11  8.7599
##                         Pr(>F)    
## verse_prop           0.0261898 *  
## f_period3            0.5270590    
## verse_prop:f_period3 0.0002327 ***
## ---
## Signif. codes:  
## 0 '***' 0.001 '**' 0.01 '*' 0.05 '.' 0.1 ' ' 1
```

The model was re parameterised to explicitly show the 3 different
linear regression lines (intercept and slope) to match the fitted lines
in S1 Fig 6.

```
# Explore the significance of the slopes
pv3a=lmer(mode~ -1 + f_period3 + verse_prop:f_period3 + (1|Author2),data=plays3)
summary(pv3a)
```

```
## Linear mixed model fit by REML. t-tests use
##   Satterthwaite's method [lmerModLmerTest]
## Formula: 
## mode ~ -1 + f_period3 + verse_prop:f_period3 + (1 | Author2)
##    Data: plays3
## 
## REML criterion at convergence: 1099
## 
## Scaled residuals: 
##     Min      1Q  Median      3Q     Max 
## -4.1602 -0.4652 -0.1503  0.3586  4.2582 
## 
## Random effects:
##  Groups   Name        Variance Std.Dev.
##  Author2  (Intercept) 0.1921   0.4383  
##  Residual             3.7922   1.9473  
## Number of obs: 263, groups:  Author2, 31
## 
## Fixed effects:
##                               Estimate Std. Error       df
## f_period3< 1597                 6.2667     0.6983  82.7869
## f_period31597-1602              5.2296     0.8103 216.9671
## f_period3> 1602                 5.4048     0.4736 177.6263
## f_period3< 1597:verse_prop      3.1031     0.8262 106.6532
## f_period31597-1602:verse_prop   1.5080     1.1756 247.5957
## f_period3> 1602:verse_prop     -1.1139     0.6118 199.2804
##                               t value Pr(>|t|)    
## f_period3< 1597                 8.974 7.52e-14 ***
## f_period31597-1602              6.454 7.00e-10 ***
## f_period3> 1602                11.411  < 2e-16 ***
## f_period3< 1597:verse_prop      3.756 0.000282 ***
## f_period31597-1602:verse_prop   1.283 0.200783    
## f_period3> 1602:verse_prop     -1.821 0.070152 .  
## ---
## Signif. codes:  
## 0 '***' 0.001 '**' 0.01 '*' 0.05 '.' 0.1 ' ' 1
## 
## Correlation of Fixed Effects:
##               f_p3<1597 f_p31597-1602 f_p3>1602 f_3<1597:
## f_p31597-1602  0.002                                     
## f_prd3>1602    0.001     0.017                           
## f_p3<1597:_   -0.929     0.005         0.019             
## f_31597-1602:  0.008    -0.920         0.003     0.009   
## f_p3>1602:_    0.002     0.016        -0.907     0.000   
##               f_31597-1602:
## f_p31597-1602              
## f_prd3>1602                
## f_p3<1597:_                
## f_31597-1602:              
## f_p3>1602:_   -0.012
```

```
pv3b=lmer(mode~ -1 + f_period3 + verse_prop + (1|Author2),data=plays3)
summary(pv3b)
```

```
## Linear mixed model fit by REML. t-tests use
##   Satterthwaite's method [lmerModLmerTest]
## Formula: mode ~ -1 + f_period3 + verse_prop + (1 | Author2)
##    Data: plays3
## 
## REML criterion at convergence: 1119.7
## 
## Scaled residuals: 
##     Min      1Q  Median      3Q     Max 
## -3.8861 -0.3808 -0.2222  0.3150  4.2572 
## 
## Random effects:
##  Groups   Name        Variance Std.Dev.
##  Author2  (Intercept) 0.3703   0.6085  
##  Residual             3.9289   1.9821  
## Number of obs: 263, groups:  Author2, 31
## 
## Fixed effects:
##                    Estimate Std. Error       df t value
## f_period3< 1597      8.2735     0.4641 115.3019  17.829
## f_period31597-1602   5.8483     0.4462 154.5584  13.107
## f_period3> 1602      4.2504     0.3953 131.6210  10.753
## verse_prop           0.5083     0.4754 195.4047   1.069
##                    Pr(>|t|)    
## f_period3< 1597      <2e-16 ***
## f_period31597-1602   <2e-16 ***
## f_period3> 1602      <2e-16 ***
## verse_prop            0.286    
## ---
## Signif. codes:  
## 0 '***' 0.001 '**' 0.01 '*' 0.05 '.' 0.1 ' ' 1
## 
## Correlation of Fixed Effects:
##             f_3<15 f_3159 f_3>16
## f_31597-160  0.614              
## f_prd3>1602  0.723  0.632       
## verse_prop  -0.791 -0.650 -0.825
```

```
# Plot the fitted relationship
# fit1 = data.frame(verse_prop=plays3$verse_prop,f_period3=plays3$f_period3,pred=predict(pv3a,re.form=NA,na.action=na.exclude))
# ggplot(fit1,aes(x=verse_prop,y=pred)) + geom_point() + facet_grid(~f_period3) + labs(title = "Plot of interaction between verse proportion and time period",x="Proportion of text in a play as verse", y="Predicted Mode")
```

For the period before 1597 there was a significant positive slope,
b(SE) = 3.1(0.83), p < .001, for 1597-1602 the slope decreased and
was no longer significant, b(SE) = 1.51(1.18), p=.20. After 1602 the
slope became weakly negative b(SE) = -1.11(0.61), p = .07.

Fitting a model with only the main effect of verse proportion gave a
non-significant slope for verse proportion (p=.29) with slope
coefficient 0,51. This means that over the full range of verse
proportions the mode would be expected to increase by 0.5 on average,
or0.25 for an increase of 0.5 in verse proportion, going from 0.40 to
0.90. Overall it was decided that the proportion of verse in a play was
not a variable worth considering further in the modelling of the
mode.

## Verse proportion by time and genre

Additional graphical exploration of verse proportion over time by
genre groupings revealed some interesting features. The two part figure
S1 Fig 7, revealed that quite a few plays in the period 1597 to 1612
(the transition period 1597 to 1602 and 1612 are shown by dotted lines)
had verse proportions below 50% and that these were predominantly
comedies. After 1612 perhaps the experiment with the less structured
form of writing declined with only a few comedies having verse
proportions less than 50%.

S1 Fig 7 Proportion of verse in a play by date (upper)and proportion of
verse in play by date, in five genre groups (lower)

The table below shows the number of comedies by each author in
categories of proportion of verse depending on whether they had less
than 50% of the text as verse (Yes) or 50% or more (No). Of those with 3
plays or more only John Fletcher (8 out of 8) wrote plays exclusively
with verse proportion above 50%, the others (with one exception Richard
Broome who�s 4 plays all had less than 50% verse proportion) had a
relatively balanced mixture of the two.

```
## [1] Table of authors who wrote plays with verse proportion less than 50% divided into comedy or other groups
```

```
##                     Author No Yes Total
## 1               Jonson Ben  8   6    14
## 2         Middleton Thomas  5   7    12
## 3      Shakespeare William  8   4    12
## 4           Chapman George  3   5     8
## 5            Fletcher John  8   0     8
## 6            Dekker Thomas  2   3     5
## 7                Uncertain  4   1     5
## 8            Brome Richard  0   4     4
## 9             Marston John  1   3     4
## 10                Day John  1   2     3
## 11          Heywood Thomas  2   1     3
## 12               Lyly John  1   2     3
## 13        Haughton William  2   0     2
## 14         Sharpham Edward  0   2     2
## 15           Shirley James  2   0     2
## 16            Armin Robert  1   0     1
## 17           Barry Lording  1   0     1
## 18        Beaumont Francis  0   1     1
## 19              Cooke John  0   1     1
## 20            Field Nathan  1   0     1
## 21               Ford John  1   0     1
## 22           Greene Robert  1   0     1
## 23      Markham and Machin  1   0     1
## 24      Marmion Shackerley  0   1     1
## 25          Munday Anthony  1   0     1
## 26      Munday Anthony (?)  1   0     1
## 27            Nashe Thomas  1   0     1
## 28            Phillip John  1   0     1
## 29            Porter Henry  1   0     1
## 30           Rowley Samuel  0   1     1
## 31 Shakespeare & Middleton  1   0     1
## 32          Udall Nicholas  1   0     1
## 33           Wilson Robert  1   0     1
## 34                     Sum 61  44   105
```

## Verse proportion for comedies only versus speech length

The final facet of this was to see if within the set of comedies
whether there was a relationship between verse proportion and the
distribution of speech length, based on the mode, median or mean. The 3
plots in S1 Fig 8, one for each summary measure, suggest there was none.
The implication from this then being that the time based changes were
not related to differences in the verse structure, but more fundamental
changes in the form of writing to make for shorter speeches.

S1 Fig 8 Comedies only: mode, median and mean of speech lengths versus
proportion of verse. The fitted line is based on a GAM smoother.

# Modelling relationships for the mode, mean and median

The primary analysis method used was to fit Linear Mixed Models
(LMMs) to explore the relationship between speech length for each play
with 3 explanatory factors of genre, performing company play type and
time (as a 3-group variable based on the cusum chart analysis above).
Three different measures of central tendency, the mode, mean and median
of each play are assessed in the sections that follow.

# Mode

The relationship between the mode and the genre, play type and time
period (in its 3 level categorical form) was explored visually in S1 Fig
9 below. The grouping Other is made up of 59 Boys Professional and 20
Miscellaneous plays, the remainder being Adult professional. Some
notable features are the absence of history plays in the play type
category Other. Tragicomedies were written mostly post 1602 and there
were quite a few miscellaneous plays. The lower modes post 1602 are
evident in many of the genre/play type combinations.

S1 Fig 9 Mode of speech length (with jittering) by genre, play type and
time groups.

Based on the analysis carried out in Supplementary 2 a LMM was fit
(normal distribution) with model weights based on an SD function to
adjust for non-constant variability in the residuals. The function
coefficients are taken from the SD function determined using in
iterative modelling scheme in Supplmentary 2. The dataset used has two
plays omitted, The Tragedy of Miriam with and extreme mode of 33 and
Philotas that had a Pearson residual of 9. As for the model variables in
the LMM author will be a random effect and genre, play type and time
period as fixed effects.

The first step was to fit a base model without observation weights.
In the output below time period and Genre were significant, p <.001
and p=.03 respectively.

```
# Base model without weights

mod0<-lmer(mode~ Genre + PlayType2 + f_period3 + (1|Author2),data=plays3,REML=F)
anova(mod0)
```

```
## Type III Analysis of Variance Table with Satterthwaite's method
##           Sum Sq Mean Sq NumDF  DenDF F value  Pr(>F)    
## Genre      39.43   9.858     4 271.33  2.6585 0.03322 *  
## PlayType2   0.02   0.018     1 185.45  0.0049 0.94408    
## f_period3 473.52 236.761     2 230.27 63.8511 < 2e-16 ***
## ---
## Signif. codes:  
## 0 '***' 0.001 '**' 0.01 '*' 0.05 '.' 0.1 ' ' 1
```

The SD function used was obtained from the iterative fitting scheme
in Supplementary 2. It was based on fitting a linear model to the
absolute value of the residuals against predicted values from the base
model, see S1 Fig 10. The linear equation in the plot illustrates the
approach but the coefficients for the line are close but not the same as
the fitted line from the iterative scheme in Supplementary 2. The SD
function coefficients used in the weighting scheme below are taken from
those obtain in Supplementary 2.

The SD function was SD = -0.2055 + 0.2458\*predicted.  
Over the range of predicted values this indicates the SD of residuals
increases from 0.78 for the lowest predicted values (4) to 2.25 for the
highest predicted values (10), a 2.9 fold increase from the lowest to
highest predicted values. The observation weights (w) were calculated as
w = 1/SD2.

```
options(width=110)
# Fit the normal model with weights based on the normal distribution model absolute residuals
plays3$w = 1/((-0.2055 + 0.2458*predict(mod0))^2)

# model using weights to adjust for non-constant variability in the residuals
mod1<-lmer(mode~ Genre + PlayType2 + f_period3 + (1|Author2),weight = w,data=plays3,REML=F)
summary(mod1)
```

```
## Linear mixed model fit by maximum likelihood . t-tests use Satterthwaite's method ['lmerModLmerTest']
## Formula: mode ~ Genre + PlayType2 + f_period3 + (1 | Author2)
##    Data: plays3
## Weights: w
## 
##      AIC      BIC   logLik deviance df.resid 
##   1094.1   1130.1   -537.0   1074.1      263 
## 
## Scaled residuals: 
##     Min      1Q  Median      3Q     Max 
## -2.7911 -0.5238 -0.1193  0.3492  3.6012 
## 
## Random effects:
##  Groups   Name        Variance Std.Dev.
##  Author2  (Intercept) 0.1269   0.3562  
##  Residual             1.9353   1.3912  
## Number of obs: 273, groups:  Author2, 31
## 
## Fixed effects:
##                     Estimate Std. Error        df t value Pr(>|t|)    
## (Intercept)          8.40056    0.37523 229.36741  22.388  < 2e-16 ***
## GenreHistory         1.15546    0.44371 272.71852   2.604  0.00972 ** 
## GenreMisc            0.16262    0.38586 266.91992   0.421  0.67376    
## GenreTragedy        -0.49971    0.23335 265.67663  -2.141  0.03315 *  
## GenreTragicomedy    -0.61216    0.30551 259.93359  -2.004  0.04614 *  
## PlayType2Other       0.08074    0.24013 141.04846   0.336  0.73717    
## f_period31597-1602  -2.56409    0.42835 271.00741  -5.986  6.8e-09 ***
## f_period3> 1602     -3.70178    0.35623 250.69778 -10.391  < 2e-16 ***
## ---
## Signif. codes:  0 '***' 0.001 '**' 0.01 '*' 0.05 '.' 0.1 ' ' 1
## 
## Correlation of Fixed Effects:
##             (Intr) GnrHst GnrMsc GnrTrgd GnrTrgc PlyT2O f_3159
## GenreHistry -0.286                                            
## GenreMisc   -0.385  0.149                                     
## GenreTragdy -0.264  0.228  0.238                              
## GenrTrgcmdy -0.184  0.179  0.163  0.359                       
## PlyTyp2Othr -0.257  0.187 -0.055  0.149   0.163               
## f_31597-160 -0.681 -0.034  0.266  0.046   0.013  -0.021       
## f_prd3>1602 -0.853  0.142  0.266 -0.039  -0.068   0.036  0.706
```

```
anova(mod1)
```

```
## Type III Analysis of Variance Table with Satterthwaite's method
##            Sum Sq Mean Sq NumDF  DenDF F value    Pr(>F)    
## Genre      34.951   8.738     4 268.78  4.5149  0.001515 ** 
## PlayType2   0.219   0.219     1 141.05  0.1131  0.737175    
## f_period3 216.033 108.016     2 260.49 55.8133 < 2.2e-16 ***
## ---
## Signif. codes:  0 '***' 0.001 '**' 0.01 '*' 0.05 '.' 0.1 ' ' 1
```

From the Type III Analysis of Variable Table the model with weights
had both time period (p <.001) and genre still significant, but more
so now, p=.002, and play type still not significant p=.74.

The estimated marginal means (EMMs) from this model for time period
were examined in the 2 tables that follow, the EMMs and the pairwise
differences between the EMMs. The EMMs indicate a progressive lowering
in the mean of the mode from the highest level before 1597, 8.5, through
the transition period 1597-1602, 5.9, and lowest for the post 1602
period, 4.8, the differences between the successive pairs of time
periods being significant, < 1597 to (1597-1602) p <.001 and
(1597-1602) to 1602 p = .001.

S1 Table 4 Estimated Marginal Means (EMM) and 95% CIs for the 3
time periods, from the final LMM

| Time Period | EMM | SE | df | LCL | UCL |
| --- | --- | --- | --- | --- | --- |
| < 1597 | 8.48 | 0.32 | 12.51 | 7.79 | 9.17 |
| 1597-1602 | 5.92 | 0.31 | 79.11 | 5.30 | 6.53 |
| > 1602 | 4.78 | 0.19 | 83.68 | 4.41 | 5.15 |

S1 Table 5 Pairwise differences in time period EMMs and significance
of the differences, from the final LMM.

```
##  contrast             estimate    SE    df t.ratio p.value
##  < 1597 - (1597-1602)     2.56 0.426  39.3   6.023  <.0001
##  < 1597 - > 1602          3.70 0.350  23.5  10.564  <.0001
##  (1597-1602) - > 1602     1.14 0.316 133.4   3.597  0.0005
## 
## Results are averaged over the levels of: Genre, PlayType2 
## Degrees-of-freedom method: kenward-roger
```

The EMMs and differences between categories for genre are examined
below in a similar fashion. The primary pattern being that historical
plays have higher modes on average than the other genres with
significant differences between history and comedy, Tragedy and
Tragicomedy, p=.01, p=.001 and p=.001 respectively. The other genres are
not significantly different to each other with none of the differences
being significant apart from comedy significantly higher than tragedy,
p=.04.

S1 Table 6 Estimated Marginal Means (EMM) and 95% CIs for the 5
genres, from the final LMM

| Genre | EMM | SE | df | LCL | UCL |
| --- | --- | --- | --- | --- | --- |
| Comedy | 6.35 | 0.21 | 29.37 | 5.93 | 6.77 |
| History | 7.51 | 0.42 | 45.91 | 6.67 | 8.35 |
| Misc | 6.51 | 0.35 | 50.95 | 5.80 | 7.23 |
| Tragedy | 5.85 | 0.25 | 47.09 | 5.35 | 6.35 |
| Tragicomedy | 5.74 | 0.32 | 130.12 | 5.10 | 6.38 |

S1 Table 7 Pairwise differences in genre EMMs and significance of the
differences, from the final LMM.

```
##  contrast              estimate    SE    df t.ratio p.value
##  Comedy - History        -1.155 0.448  61.4  -2.577  0.0124
##  Comedy - Misc           -0.163 0.389  78.9  -0.418  0.6767
##  Comedy - Tragedy         0.500 0.239 259.4   2.088  0.0378
##  Comedy - Tragicomedy     0.612 0.313 386.8   1.953  0.0515
##  History - Misc           0.993 0.544  52.0   1.825  0.0737
##  History - Tragedy        1.655 0.456  66.5   3.627  0.0006
##  History - Tragicomedy    1.768 0.497  86.8   3.555  0.0006
##  Misc - Tragedy           0.662 0.405  85.3   1.634  0.1060
##  Misc - Tragicomedy       0.775 0.457 114.9   1.696  0.0926
##  Tragedy - Tragicomedy    0.112 0.317 435.2   0.354  0.7232
## 
## Results are averaged over the levels of: PlayType2, f_period3 
## Degrees-of-freedom method: kenward-roger
```

The EMMs for each of the fixed effects are shown in S1 Fig 10. The
same scale across each plot allows visual assessment of the relative
effects sizes associated with each variable. The differences for time
period are much larger than for genre which in turn was larger than for
play type (which was not significant). Within each plot letters have
been added to show which levels are statistically different. They were
assigned based on EMM pairwise t tests between the levels (unadjusted
for multiple comparisons). Categories with different letters are
significantly different. However in the genre plot the pattern of
pairwise significance differences did not lead to a consistent letter
pattern at the .05 significance level that could be simply expressed in
the plot. Therefore for simplicity of interpretation the assignment of
letters in the genre plot figure was based on the .11 significance
level. Historical plays had the highest mode at about 7.5, being higher
than the second tier group of comedy and miscellaneous at about 6.4 (b)
with tragedy and tragicomedy being similar at the lowest level of about
5.8 (c).

S1 Fig 10 Mode of speech length, EMMs and 95% CIs from the final LMM
model for each of the explanatory variables, genre, play type and time
period. Within each plot letters have been added to show which levels
are statistically different, categories with different letters are
different.

### Checking model assumptions - residuals and random effects

The reliability of model estimates is influenced by how well model
assumptions are met. Residual diagnostics and the distribution of the
random effects are checked below.

#### Residuals

The distribution of residuals for the final model (S1 Fig 11) are
slightly right skewed, but overall the histogram distributions appears
symmetric enough that the benefits of the central limit theorem should
protect the reliability of the uncertainties on the means. The histogram
of the Pearson residuals shows a small group of plays that are more than
2, i.e.�2 SDs, higher than the model prediction. These do not fit the
model quite so well due to some other factor perhaps. The normal Q-Q
(quantile) plots, unscaled and Pearson residuals, show more clearly than
the histograms how the upper tail of the distribution has the largest
departure from normality.

S1 Fig 11 Residual diagnostics for the final model for the mode

#### Random effects

The assumption of normality of the random effects for the LMM is met
well, see the two right hand graphs in S1 Fig 12, the normal quantile
plot and histogram. The caterpillar plot in the left had side of S1 Fig
12 shows the random effects, namely the estimated means (Best Linear
Unbiased Prediction, BLUP) for each author along with their 95% CIs. The
random effects range from the lowest level of about -0.3 for the author
with the lowest mode, John Marston, to the highest of about +0.4 for the
uncertain author group. Comparing the size of the author differences,
0.7 from lowest to highest, with the change over time of 3.7 (8.5 down
to 4.8) shows that the the impact of the event(s) that led to a general
decrease in mode for plays before and after 1600 had a much larger
effect than individual author differences.

As an example to help understand the author random effects, consider
the predicted mean modal value for plays after 1602. Without
consideration of authorship, that is, averaged over all authors, the EMM
would be 4.8. If however predictions were to be made for specific
authors in this period, then for John Marston it would be 4.8 - 0.3 =
4.5 and for Richard Broome 4.8 + 0.4 = 5.2.

S1 Fig 12 Distribution of author random effects for the mode.

```
##                      Author   BLUP se_BLUP
## 1              Marston John -0.312   0.279
## 2                 Lyly John -0.296   0.328
## 3       Shakespeare William -0.272   0.225
## 4             Greene Robert -0.197   0.343
## 5                 Ford John -0.175   0.279
## 6                Jonson Ben -0.158   0.237
## 7             Fletcher John -0.116   0.224
## 8           Sharpham Edward -0.103   0.332
## 9            Heywood Thomas -0.092   0.265
## 10 Shakespeare and Fletcher -0.087   0.332
## 11            Dekker Thomas -0.068   0.309
## 12            Rowley Samuel -0.060   0.336
## 13  Shakespeare & Middleton -0.060   0.330
## 14       Munday and Chettle -0.058   0.346
## 15   Shakespeare and others -0.055   0.348
## 16         Beaumont Francis -0.038   0.334
## 17           Chapman George -0.035   0.270
## 18         Massinger Philip -0.031   0.327
## 19    Beaumont and Fletcher  0.023   0.304
## 20               Kyd Thomas  0.024   0.346
## 21            Shirley James  0.024   0.278
## 22            Wilson Robert  0.038   0.347
## 23             Webster John  0.111   0.317
## 24             Peele George  0.118   0.344
## 25                 Day John  0.145   0.326
## 26         Haughton William  0.178   0.344
## 27      Marlowe Christopher  0.189   0.341
## 28         Middleton Thomas  0.230   0.237
## 29       Single Play Author  0.361   0.207
## 30            Brome Richard  0.366   0.308
## 31                Uncertain  0.406   0.276
```

### About interactions for the mode - none significant

```
# The main effects model reported above was the best choice, none of the interactions were significant
# These 2 models here are recorded to confirm this
# For the 2 significant main effects in the model genre and time period the 2 way interaction was not significant, output not shown, p=.76
mod2<-lmer(mode~ Genre + PlayType2 + f_period3 + Genre:f_period3 + (1|Author2),weight = w,data=plays3,REML=F)
anova(mod2)
```

```
## Type III Analysis of Variance Table with Satterthwaite's method
##                  Sum Sq Mean Sq NumDF  DenDF F value    Pr(>F)    
## Genre            23.118   5.779     4 264.99  3.0515   0.01752 *  
## PlayType2         0.038   0.038     1 147.35  0.0199   0.88795    
## f_period3       122.393  61.197     2 272.48 32.3110 2.557e-13 ***
## Genre:f_period3  10.022   1.253     8 264.29  0.6614   0.72527    
## ---
## Signif. codes:  0 '***' 0.001 '**' 0.01 '*' 0.05 '.' 0.1 ' ' 1
```

```
# Nor any in  the full factorial model - but this model is useful for comparison with the model for the mean below where there were significant interactions
# None of the unusual combinations that were evident in the mean we found for the mode (see the comparison below in the section on the mean)
mod3<-lmer(mode ~ Genre * f_period3 * PlayType2 + (1|Author2),weight = w,data=plays3)
```

```
## fixed-effect model matrix is rank deficient so dropping 5 columns / coefficients
```

```
anova(mod3)
```

```
## Missing cells for: GenreHistory:PlayType2Other, GenreMisc:f_period31597-1602:PlayType2Adult Professional, GenreTragicomedy:f_period31597-1602:PlayType2Adult Professional, GenreHistory:f_period3< 1597:PlayType2Other, GenreHistory:f_period31597-1602:PlayType2Other, GenreHistory:f_period3> 1602:PlayType2Other.  
## Interpret type III hypotheses with care.
```

```
## Type III Analysis of Variance Table with Satterthwaite's method
##                            Sum Sq Mean Sq NumDF  DenDF F value    Pr(>F)    
## Genre                      18.271   4.568     4 242.50  2.2424   0.06513 .  
## f_period3                 112.123  56.062     2 246.17 27.5220 1.632e-11 ***
## PlayType2                   0.578   0.578     1 237.44  0.2836   0.59484    
## Genre:f_period3            12.025   1.503     8 241.80  0.7379   0.65793    
## Genre:PlayType2            10.794   3.598     3 241.78  1.7664   0.15420    
## f_period3:PlayType2         0.325   0.162     2 244.80  0.0797   0.92338    
## Genre:f_period3:PlayType2   1.890   0.473     4 241.74  0.2320   0.92021    
## ---
## Signif. codes:  0 '***' 0.001 '**' 0.01 '*' 0.05 '.' 0.1 ' ' 1
```

### Shakespeare compared with all other authors

The right hand side panel in S1 Fig 13 for Shakespeare shows that his
work moved to shorter modes during and after the transition period
1597-1602 in line with the general trend observed for plays for all
other authors in the left hand panel.

S1 Fig 13 The modes for speech lengths for Shakespeare plays and for
plays by others, with a smoothed line, and the 95% confidence band. The
range for Shakespeare plays is 1590 to 1613; the range for plays by
other authors is 1538 to 1642. The 1597 and 1602 boundaries are shown as
dotted vertical lines.

# Analysis using only adult professional play types

A model for the mode with only the adult professional plays was
fitted for comparison with an equivalent model in the next section on
the analysis of the mean. As the interaction between genre and time
period was not significant the model was simplified to keeping only main
effects.

```
# Data set only has Adult professional - the interaction term is not significant
mod_ap1<-lmer(mode~ Genre * f_period3 + (1|Author2),weight = w,data=plays_ap)
anova(mod_ap1)
```

```
## Type III Analysis of Variance Table with Satterthwaite's method
##                  Sum Sq Mean Sq NumDF  DenDF F value    Pr(>F)    
## Genre            29.234   7.309     4 179.50  3.5884  0.007672 ** 
## f_period3       102.320  51.160     2 166.63 25.1189 2.919e-10 ***
## Genre:f_period3  11.079   1.846     6 176.92  0.9066  0.491464    
## ---
## Signif. codes:  0 '***' 0.001 '**' 0.01 '*' 0.05 '.' 0.1 ' ' 1
```

```
# So simplify with a main effects model
mod_ap2<-lmer(mode~ Genre + f_period3 + (1|Author2),weight = w,data=plays_ap)
anova(mod_ap2)
```

```
## Type III Analysis of Variance Table with Satterthwaite's method
##            Sum Sq Mean Sq NumDF   DenDF F value    Pr(>F)    
## Genre      33.165   8.291     4 178.708  4.0476  0.003631 ** 
## f_period3 174.109  87.054     2  92.043 42.4981 8.439e-14 ***
## ---
## Signif. codes:  0 '***' 0.001 '**' 0.01 '*' 0.05 '.' 0.1 ' ' 1
```

## Mean

As the distribution of speech length for most plays is right skewed,
approximating that of a log normal distribution (not shown in this
supplementary material analysis, but a feeling for it can be obtained
from the histograms in S1 Fig 4). The mean as a measure of central
tendency would be more sensitive to plays with a high proportion of
longer speeches than the mode or the median.

In S1 Fig 14 the play means for combinations of genre, play type and
time period are shown. The absence of historical plays for the Other
play type category is evident along with lower numbers of plays in the
Other category (79/273 = 29%). Also the large spread in means for the
Other play type for tragedies for the period prior to 1597 is noted.

S1 Fig 14 Mean speech length of plays in genre, play type and time
period groupings

There were 9 plays with means greater than 70 (listed below), 8 out
of the 9 being prior to 1597.

```
##                          TextTitle           Author       Genre          PlayType2 f_period3  Mean
## 1                Ferrex and Porrex Sackville Thomas     Tragedy              Other    < 1597 137.7
## 2                        Cleopatra    Daniel Samuel     Tragedy              Other    < 1597 111.6
## 3                 Virtuous Octavia   Brandon Samuel Tragicomedy              Other 1597-1602  89.7
## 4              Tancred and Gismund    Wilmot et al.     Tragedy              Other    < 1597  80.2
## 5 Summer's Last Will and Testament     Nashe Thomas      Comedy              Other    < 1597  77.1
## 6                         Cornelia       Kyd Thomas     Tragedy              Other    < 1597  75.8
## 7                         Antonius      Sidney Mary     Tragedy              Other    < 1597  75.5
## 8                        1 Selimus        Uncertain        Misc Adult Professional    < 1597  74.4
## 9                Battle of Alcazar     Peele George        Misc Adult Professional    < 1597  72.7
```

A full factorial LMM was fitted to examine all combinations of genre,
play type and time period. All main effect and interaction terms were
significant suggesting a complex interpretation. This was not the case
with mode, where none of the interactions were significant confirming
the greater sensitivity of the mean to the right skewed distributions.
The model EMMs and CIs for the mean are plotted in upper plot of S1 Fig
15 with the comparison with the EMMs for the mode from the equivalent
model below it. Apart from the missing combinations the most notable
feature was in the Other play type category where there were two
instances of substantially higher means, for Tragedy prior to 1597 and
Tragicomedy in the 1597-1602 period. This suggests the adult
professional play type was different in some way to the other types in
those genres.

```
## Type III Analysis of Variance Table with Satterthwaite's method
##                           Sum Sq Mean Sq NumDF DenDF F value    Pr(>F)    
## Genre                     3642.4  910.60     4   248 10.1391 1.285e-07 ***
## f_period3                 2250.7 1125.35     2   248 12.5302 6.545e-06 ***
## PlayType2                  573.1  573.08     1   248  6.3810  0.012158 *  
## Genre:f_period3           4442.9  555.36     8   248  6.1837 2.779e-07 ***
## Genre:PlayType2           3470.1 1156.71     3   248 12.8794 7.562e-08 ***
## f_period3:PlayType2       1130.3  565.15     2   248  6.2927  0.002159 ** 
## Genre:f_period3:PlayType2 4891.8 1222.94     4   248 13.6169 4.739e-10 ***
## ---
## Signif. codes:  0 '***' 0.001 '**' 0.01 '*' 0.05 '.' 0.1 ' ' 1
```

S1 Fig 15 Relationship bewtween Mean speech length and genre, play type
and time period. EMMs and 95% CIs from the full factorial LMM model

For a better comparison between genres an analysis was carried out
excluding the Other play type category so that only Adult Professional
play types were compared.

```
# With only adult professional plays the interaction between time and genre became non significant, p=.09
m2a<-lmer(length_mean ~ Genre * f_period3  + (1|Author2),data=plays_ap)
anova(m2a)
```

```
## Type III Analysis of Variance Table with Satterthwaite's method
##                  Sum Sq Mean Sq NumDF DenDF F value    Pr(>F)    
## Genre           1467.09  366.77     4   181  8.2294 4.042e-06 ***
## f_period3        386.06  193.03     2   181  4.3311   0.01454 *  
## Genre:f_period3  504.23   84.04     6   181  1.8856   0.08550 .  
## ---
## Signif. codes:  0 '***' 0.001 '**' 0.01 '*' 0.05 '.' 0.1 ' ' 1
```

```
# This meant a model simplification was reasonable - the main effects model would provide a simpler comparison between genres
m3<-lmer(length_mean ~ Genre + f_period3  + (1|Author2),data=plays_ap)
anova(m3)
```

```
## Type III Analysis of Variance Table with Satterthwaite's method
##            Sum Sq Mean Sq NumDF DenDF F value    Pr(>F)    
## Genre     2066.14  516.54     4   187 11.2695 3.257e-08 ***
## f_period3  900.12  450.06     2   187  9.8191 8.810e-05 ***
## ---
## Signif. codes:  0 '***' 0.001 '**' 0.01 '*' 0.05 '.' 0.1 ' ' 1
```

#### EMMs and significance of differences for the mean model

```
# Marginal means and significance of differences between them
emmeans(m3, ~ f_period3)
```

```
##  f_period3 emmean   SE   df lower.CL upper.CL
##  < 1597      31.6 1.07 38.0     29.5     33.8
##  1597-1602   27.5 1.38 75.5     24.8     30.2
##  > 1602      25.8 0.78 42.6     24.2     27.4
## 
## Results are averaged over the levels of: Genre 
## Degrees-of-freedom method: kenward-roger 
## Confidence level used: 0.95
```

```
# Difference in means - with p values
pairs(emmeans(m3, ~ f_period3),adjust="none")
```

```
##  contrast             estimate   SE    df t.ratio p.value
##  < 1597 - (1597-1602)     4.12 1.63 187.0   2.532  0.0122
##  < 1597 - > 1602          5.83 1.38  90.4   4.228  0.0001
##  (1597-1602) - > 1602     1.71 1.54 124.0   1.110  0.2693
## 
## Results are averaged over the levels of: Genre 
## Degrees-of-freedom method: kenward-roger
```

```
emmeans(m3, ~ Genre)
```

```
##  Genre       emmean    SE    df lower.CL upper.CL
##  Comedy        23.6 0.937  44.8     21.7     25.4
##  History       30.3 1.240  83.7     27.8     32.8
##  Misc          34.6 1.552 123.4     31.5     37.6
##  Tragedy       26.7 1.130  59.0     24.4     28.9
##  Tragicomedy   26.4 1.758 132.9     23.0     29.9
## 
## Results are averaged over the levels of: f_period3 
## Degrees-of-freedom method: kenward-roger 
## Confidence level used: 0.95
```

```
# Difference in means - with p values
pairs(emmeans(m3, ~ Genre),adjust="none")
```

```
##  contrast              estimate   SE  df t.ratio p.value
##  Comedy - History        -6.749 1.56 166  -4.322  <.0001
##  Comedy - Misc          -10.994 1.84 154  -5.981  <.0001
##  Comedy - Tragedy        -3.121 1.30 179  -2.408  0.0171
##  Comedy - Tragicomedy    -2.870 1.82 182  -1.581  0.1157
##  History - Misc          -4.245 1.93 186  -2.201  0.0290
##  History - Tragedy        3.628 1.68 181   2.160  0.0321
##  History - Tragicomedy    3.879 2.17 185   1.787  0.0756
##  Misc - Tragedy           7.873 1.91 176   4.128  0.0001
##  Misc - Tragicomedy       8.124 2.35 174   3.457  0.0007
##  Tragedy - Tragicomedy    0.251 1.89 183   0.133  0.8941
## 
## Results are averaged over the levels of: f_period3 
## Degrees-of-freedom method: kenward-roger
```

#### EMMs and significance of differences for the equivalent mode model

```
# Models for the mode
# Time period
# Marginal means and significance of differences between them
emmeans(mod_ap2, ~ f_period3)
```

```
##  f_period3 emmean    SE   df lower.CL upper.CL
##  < 1597      8.24 0.307 14.0     7.58     8.90
##  1597-1602   5.67 0.326 52.9     5.02     6.32
##  > 1602      4.83 0.198 31.4     4.43     5.23
## 
## Results are averaged over the levels of: Genre 
## Degrees-of-freedom method: kenward-roger 
## Confidence level used: 0.95
```

```
# Difference in means - with p values
pairs(emmeans(mod_ap2, ~ f_period3),adjust="none")
```

```
##  contrast             estimate    SE    df t.ratio p.value
##  < 1597 - (1597-1602)    2.570 0.419  78.1   6.139  <.0001
##  < 1597 - > 1602         3.409 0.375  41.6   9.101  <.0001
##  (1597-1602) - > 1602    0.839 0.341 135.3   2.458  0.0152
## 
## Results are averaged over the levels of: Genre 
## Degrees-of-freedom method: kenward-roger
```

```
# Genre
emmeans(mod_ap2, ~ Genre)
```

```
##  Genre       emmean    SE    df lower.CL upper.CL
##  Comedy        6.03 0.209  43.5     5.61     6.45
##  History       7.41 0.346  40.4     6.71     8.11
##  Misc          6.42 0.486  25.8     5.42     7.42
##  Tragedy       5.81 0.282  38.5     5.24     6.38
##  Tragicomedy   5.56 0.394 106.3     4.77     6.34
## 
## Results are averaged over the levels of: f_period3 
## Degrees-of-freedom method: kenward-roger 
## Confidence level used: 0.95
```

```
# Difference in means - with p values
pairs(emmeans(mod_ap2, ~ Genre),adjust="none")
```

```
##  contrast              estimate    SE    df t.ratio p.value
##  Comedy - History        -1.382 0.402  76.1  -3.434  0.0010
##  Comedy - Misc           -0.394 0.536  39.8  -0.736  0.4660
##  Comedy - Tragedy         0.216 0.288 148.5   0.752  0.4535
##  Comedy - Tragicomedy     0.471 0.385 192.0   1.224  0.2225
##  History - Misc           0.987 0.584  39.5   1.691  0.0988
##  History - Tragedy        1.598 0.442  76.6   3.616  0.0005
##  History - Tragicomedy    1.853 0.524 101.8   3.538  0.0006
##  Misc - Tragedy           0.611 0.559  43.0   1.093  0.2804
##  Misc - Tragicomedy       0.866 0.626  54.9   1.383  0.1724
##  Tragedy - Tragicomedy    0.255 0.426 151.8   0.598  0.5504
## 
## Results are averaged over the levels of: f_period3 
## Degrees-of-freedom method: kenward-roger
```

In S1 Fig 16 the patterns of the EMMs are for the adult professional
play types only. The genre and time period effects are compared for the
two summary measures mean and the mode. The most obvious difference
between them is that for the mode the time period effect is the biggest
impact, however for the mean genre is more important having larger
differences. Using the uncertainty in the EMMs as shown by the CIs the
separation between the EMMs for comedy and history and miscellaneous
genres is larger in the mean than for the mode. Indeed for the mean the
miscellaneous group had a significantly higher EMM than history, for the
mode they were the same. In addition for the mean comedy had the
shortest EMM speech length being significantly lower to all genres with
the exception of tragicomedy, for the mode the tragedy and tragicomedy
genres have lower modes than comedy although not significantly
different. These differences are attributed to the greater sensitivity
of the mean for right skewed distributions where longer speeches are
more influential on the mean than they are for the mode (or the median).
The mean and mode provide complementary views of the play speech length
distributions and so each contributes to increased understanding of
differences between the genres. For example with the EMM mean for comedy
being lower than all the others it suggests that all speeches, short and
long, were generally kept shorter in comedies compared to other genres
for plays produced by adult professional companies. The time period
effect is shown more dramatically with the mode with the difference
between the transition period 1597-1602 and after 1602 being more
obvious than with the mean.

S1 Fig 16 Comparison of the mean and mode for the genre and time period
effects for Adult Professional play types only. EMMs and 95% CIs are
from the main effect only models. Within each plot letters have been
added to show which levels are statistically different, categories with
different letters are different, when letters are shared between groups
the differences are not large enough to achieve significance.

### Mean - Diagnostics and random effect for differences between authors

The residuals for the main effects model with the mean are somewhat
right skewed but still deemed satisfactory. Two large positive Pearson
residuals of about 5 suggest a couple of plays don�t fit then model so
well.

S1 Fig 17 Model diagnostics for the mean

The standard deviation for the author random effect was zero for the
final model for the mean so there are no author differences for the mean
- they are all the same. So there is no point in making a random effects
plot.

# Median

A plot of the medians in play type and genre groupings is provided in
S1 Fig 18.

The following main effects only model with the median has been run to
match that for the mean, the pattern of medians being similar to that of
the mean.

```
med3<-lmer(length_median ~ Genre + f_period3  + (1|Author2),data=plays_ap)
summary(med3)
```

```
## Linear mixed model fit by REML. t-tests use Satterthwaite's method ['lmerModLmerTest']
## Formula: length_median ~ Genre + f_period3 + (1 | Author2)
##    Data: plays_ap
## 
## REML criterion at convergence: 1130
## 
## Scaled residuals: 
##     Min      1Q  Median      3Q     Max 
## -2.0326 -0.4836 -0.0609  0.3430  5.6069 
## 
## Random effects:
##  Groups   Name        Variance Std.Dev.
##  Author2  (Intercept)  1.317   1.148   
##  Residual             20.775   4.558   
## Number of obs: 194, groups:  Author2, 26
## 
## Fixed effects:
##                    Estimate Std. Error       df t value Pr(>|t|)    
## (Intercept)         15.7425     0.9598  96.1420  16.401  < 2e-16 ***
## GenreHistory         3.6260     1.0658 178.9493   3.402 0.000825 ***
## GenreMisc            6.1341     1.2313 185.0052   4.982 1.44e-06 ***
## GenreTragedy         0.4085     0.8867 184.4723   0.461 0.645531    
## GenreTragicomedy     0.8401     1.2418 186.0115   0.676 0.499564    
## f_period31597-1602  -3.0424     1.1146 186.9640  -2.730 0.006946 ** 
## f_period3> 1602     -3.6305     0.9629 104.0766  -3.770 0.000271 ***
## ---
## Signif. codes:  0 '***' 0.001 '**' 0.01 '*' 0.05 '.' 0.1 ' ' 1
## 
## Correlation of Fixed Effects:
##             (Intr) GnrHst GnrMsc GnrTrgd GnrTrgc f_3159
## GenreHistry -0.469                                     
## GenreMisc   -0.585  0.359                              
## GenreTragdy -0.348  0.332  0.306                       
## GenrTrgcmdy -0.188  0.193  0.180  0.313                
## f_31597-160 -0.515 -0.032  0.310  0.030   0.043        
## f_prd3>1602 -0.730  0.227  0.358 -0.062  -0.143   0.497
```

```
anova(med3)
```

```
## Type III Analysis of Variance Table with Satterthwaite's method
##           Sum Sq Mean Sq NumDF  DenDF F value    Pr(>F)    
## Genre     630.84  157.71     4 183.21  7.5915 1.111e-05 ***
## f_period3 315.48  157.74     2 148.02  7.5928 0.0007259 ***
## ---
## Signif. codes:  0 '***' 0.001 '**' 0.01 '*' 0.05 '.' 0.1 ' ' 1
```

```
# Marginal means and significance of differences between them
emmeans(med3, ~ f_period3)
```

```
##  f_period3 emmean    SE    df lower.CL upper.CL
##  < 1597      17.9 0.800  57.1     16.3     19.5
##  1597-1602   14.9 0.990 108.4     12.9     16.9
##  > 1602      14.3 0.609  38.5     13.1     15.5
## 
## Results are averaged over the levels of: Genre 
## Degrees-of-freedom method: kenward-roger 
## Confidence level used: 0.95
```

```
pairs(emmeans(med3, ~ f_period3),adjust="none")
```

```
##  contrast             estimate    SE  df t.ratio p.value
##  < 1597 - (1597-1602)    3.042 1.121 187   2.715  0.0073
##  < 1597 - > 1602         3.631 0.986 123   3.682  0.0003
##  (1597-1602) - > 1602    0.588 1.071 169   0.549  0.5837
## 
## Results are averaged over the levels of: Genre 
## Degrees-of-freedom method: kenward-roger
```

```
emmeans(med3, ~ Genre)
```

```
##  Genre       emmean    SE    df lower.CL upper.CL
##  Comedy        13.5 0.698  57.0     12.1     14.9
##  History       17.1 0.896 101.0     15.4     18.9
##  Misc          19.7 1.095 138.5     17.5     21.8
##  Tragedy       13.9 0.822  84.5     12.3     15.6
##  Tragicomedy   14.4 1.238 155.3     11.9     16.8
## 
## Results are averaged over the levels of: f_period3 
## Degrees-of-freedom method: kenward-roger 
## Confidence level used: 0.95
```

```
pairs(emmeans(med3, ~ Genre),adjust="none")
```

```
##  contrast              estimate    SE  df t.ratio p.value
##  Comedy - History        -3.626 1.080 182  -3.358  0.0010
##  Comedy - Misc           -6.134 1.249 186  -4.912  <.0001
##  Comedy - Tragedy        -0.409 0.897 185  -0.456  0.6492
##  Comedy - Tragicomedy    -0.840 1.255 186  -0.669  0.5042
##  History - Misc          -2.508 1.316 187  -1.905  0.0583
##  History - Tragedy        3.217 1.148 187   2.803  0.0056
##  History - Tragicomedy    2.786 1.485 187   1.875  0.0623
##  Misc - Tragedy           5.726 1.291 187   4.434  <.0001
##  Misc - Tragicomedy       5.294 1.602 187   3.306  0.0011
##  Tragedy - Tragicomedy   -0.432 1.293 187  -0.334  0.7391
## 
## Results are averaged over the levels of: f_period3 
## Degrees-of-freedom method: kenward-roger
```

# Comparing the patterns for the mean, median and mode

Although the median is typically intermediate in position between the
mode and the mean for skewed distributions, fitting the equivalent main
effects model to that for the mean gave very similar results. The
statistical significance and EMM patterns were very similar to that of
the mean, see S1 Fig 19.

S1 Fig 19 Comparison of main effect models for median and mean speech
length of plays in genre and time period groupings for adult
professional plays

`
