# Supplementary material for: Changes in the length of speeches in the plays of William Shakespeare and his contemporaries: A mixed models approach
Source: PLoS One. 2023 Apr 21;18(4):e0282716. doi: 10.1371/journal.pone.0282716 (PMC10121026; doi:10.1371/journal.pone.0282716)
Supplement: S2 File — (HTML) [file pone.0282716.s002.html]

S2-Model-comparison-Normal-and-Poisson.knit


### Changes in the length of speeches in the plays of William Shakespeare and his contemporaries: a mixed models approach

Colyvas, Craig and Egan

# Supplementary 2 - Comparison of Normal and Poisson Models

The analysis in this supplementary file contains a comparison of
modelling the mode using error distributions based on (a) the normal
distribution and (b) Poisson distribution using both identity and log
link functions. This arose from a suggestion by an editor that the
Poisson distribution might be more appropriate than the normal
distribution.

# Summary

From this analysis it was determined that a normal distribution model
with an appropriate weighting function was a substantially better fit
(AIC 1094) to the mode data, AIC 58 lower than a Poisson model (AIC
1152). Therefore the normal model will be used for the mode analysis
with an SD function based on the iterative scheme without the play
Philotas.

The Poisson distribution is often appropriate when working with
counted data and as speech length is a count of words it might be a
suitable error distribution for modelling the mode of the distribution
of speech lengths within a play. Poisson based models were compared to
the normal distribution model originally developed and found to be a
substantially better fit as judged by the AIC goodness of fit measure.
An investigation was carried out to understand why. This revealed 2 key
reasons for the superiority of the Poisson model.  
(a) The residual variation was not constant, increasing with larger
predicted values. The variance function inherent to the Poisson model
approximately matched the pattern giving it an advantage over the normal
distribution model where the residual variance is modeled as a constant.
(b) An outlier play with mode 33 was very influential, adversely
affecting the goodness of fit for the normal model more than the Poisson
model.

Removing the outlier play (justified on both statistical and literary
grounds, see below) and adding a weighting function to the normal
distribution model, to adjust for the non-constant variability, led to
the normal model being a a better fit than the Poisson model. This was
because the inherent Poisson SD function was much larger than required
being on average twice as large as the actual variability present in the
residuals. Therefore by using an SD function matching the variability in
the residuals the normal distribution model was a better fit than the
Poisson model. The SD function was included in the model using
observation weights calculated as 1/SD2 where SD was a linear
function of absolute residuals against predicted values, see the code,
output and explanatory text below for the details.

An attempt to determine the optimal weighting function using an
iterative scheme failed to converge displaying oscillatory behaviour. A
single play, Philotas, having a very large Pearson residual, 9 SDs, was
identified as likely very influential in causing this oscillatory
behaviour. On removal of that play the iterative scheme converged. The
final model was based on the SD function resulting from this analysis.
The normal distribution model had now become an even better fit (AIC
1094) than a Poisson model (AIC 1152) with the AIC now lower by 58.

**Conclusion**  
A model using the normal error distributon with an appropriate SD
function to correct for non-constant variance via observation weights
was clearly better than the alternative Poisson model. Two plays were
identified as outliers and need to be removed from the dataset for
reliable analysis.

### About the two plays excluded from analysis

**The Tragedy of Mariam**  
With a mode of 33 it was removed on statistical grounds, however there
were also literary grounds to support leaving Elizabeth Cary’s play ‘The
Tragedy of Mariam’ out of the analysis. This uniqueness sheds some light
on the play that literary historians would want to hear about. The play
was already an ‘outsider’ in literary history, being, almost uniquely
for this period, written by a woman. Cary was also an aristocrat, and
this distances her still further from the commercial theatre of London
in the late 16th and early 17th centuries. Thus there was already ‘prima
facie’ evidence to treat ‘The Tragedy of Mariam’ as unlike all the other
plays in this dataset, since it was not written by a professional (or
even a well-informed amateur) for intended performance in the commercial
theatres. Rather, it seems to have been written as an intellectual
exercise by a highly educated aristocratic woman who moved in a narrow
circle of aristocratic patrons of the arts, and with its intended
consumption being not in public performance but as a reading text for
her equally artistic and educated friends. Hence it is not surprising
that it doesn’t show the trends found in plays written by professional
dramatists for performance on the London stages.

**Philotas**  
This Daniel Samuel play Was excluded from the second phase of analysis
on statistical grounds due to its Pearson residual or 8.9, i.e. it was
8.9 SDs higher than the model prediction, much higher than the next
poorly fitting play with Pearson residual of 4.5. There are not strong
literary grounds for its exclusion, however its still was somewhat
unusual. The first three acts were written in 1600 and then the author
stopped writing. He took it up again in 1604 and completed the play by
writing the last two acts. When the play was first performed, in 1604,
it got the author into a lot of trouble because of its political
content.

# Compare Poisson to Normal distribution for modelling the mode

The original analysis was with a Linear Mixed Model (LMM) using the
normal distribution to model the residual error variability. Here this
is compared with an alternative, a generalised linear mixed model (GLMM)
using the Poisson distribution. The primary choice for the Poisson
distribution was using the identity link function rather than the
default canonical log link function so that coefficients and estimated
marginal means would be comparable with the normal distribution model.
For the log link function coefficients the calculation of predicted
means requires an exponential transformation from the log scale to
convert back to the original scale of the data. The comparison of means
between 2 levels of a categorical explanatory variable like genre would
then have to be assessed as the ratio of 2 means rather than a
difference for a significance test to be associated with the comparison.
However, the Poisson model with the log link was tested also for
goodness of fit but found no better than that with the identity link
function. Therefore because of the simpler comparison with the normal
distribution model the Poisson model with identity link was used in
preference.

Note that a number of functions have been written to simplify various
analyses are not shown in this output. The reader wanting more
information should check the original Rmd code files in the
supplementary files collection, the functions covering diagnostic plots
for residuals and random effects, overdspersion test for Poisson models,
fitting of SD functions and an iterative scheme to determine the best SD
function.

```
# Model using full set of potential variables
mod0<-lmer(mode~ Genre + PlayType2 + f_period3 + verse_grp + (1|Author2),data=plays,REML = F)
#summary(mod0)
anova(mod0)
```

```
## Type III Analysis of Variance Table with Satterthwaite's method
##           Sum Sq Mean Sq NumDF  DenDF F value    Pr(>F)    
## Genre      40.98  10.245     4 258.29  1.4329    0.2235    
## PlayType2  17.02  17.021     1 138.09  2.3807    0.1251    
## f_period3 414.10 207.050     2 205.55 28.9594 8.311e-12 ***
## verse_grp  41.76  13.919     3 221.38  1.9469    0.1229    
## ---
## Signif. codes:  0 '***' 0.001 '**' 0.01 '*' 0.05 '.' 0.1 ' ' 1
```

```
# Poisson equivalent of the LMM
# Because drop1 fails due to missing values drop any record with a missing among the variables used
# If drop1 is not used the original data set can be used as with either version of the data set the number of subjects and authors is the same
plays_s = na.omit(select(plays,mode, Genre, PlayType2, f_period3, verse_grp, Author2))

# Identity link function model
# The Play type variable is weakly significant but becomes non-significant in the next step 
# when the model is simplified a little by dropping the non-significant verse group variable
mod0p<-glmer(mode~ Genre + PlayType2 + f_period3 + verse_grp + (1|Author2),family=poisson(link="identity"),data=plays_s)
#summary(mod0p)
drop1(mod0p,test="Chisq")
```

```
## Single term deletions
## 
## Model:
## mode ~ Genre + PlayType2 + f_period3 + verse_grp + (1 | Author2)
##           npar    AIC    LRT   Pr(Chi)    
## <none>         1206.1                     
## Genre        4 1205.5  7.442   0.11430    
## PlayType2    1 1208.0  3.912   0.04793 *  
## f_period3    2 1251.6 49.517 1.768e-11 ***
## verse_grp    3 1205.5  5.372   0.14648    
## ---
## Signif. codes:  0 '***' 0.001 '**' 0.01 '*' 0.05 '.' 0.1 ' ' 1
```

```
# Check for overdispersion
# ratio = 1.16, i.e. the residual variance is 16% greater than would be expected for the Poisson distribution
# This is slight so consider this as satisfactory
overdisp_fun(mod0p)
```

```
##       chisq       ratio         rdf           p 
## 283.9500159   1.1223321 253.0000000   0.0880543
```

```
# Log link Poisson model
mod0pl<-glmer(mode~ Genre + PlayType2 + f_period3 + verse_grp + (1|Author2),family=poisson(link="log"),data=plays_s)
#summary(mod0pl)
drop1(mod0pl,test="Chisq")
```

```
## Single term deletions
## 
## Model:
## mode ~ Genre + PlayType2 + f_period3 + verse_grp + (1 | Author2)
##           npar    AIC    LRT   Pr(Chi)    
## <none>         1207.7                     
## Genre        4 1206.4  6.706    0.1523    
## PlayType2    1 1208.3  2.638    0.1043    
## f_period3    2 1247.1 43.412 3.742e-10 ***
## verse_grp    3 1207.5  5.788    0.1224    
## ---
## Signif. codes:  0 '***' 0.001 '**' 0.01 '*' 0.05 '.' 0.1 ' ' 1
```

```
# Overdispersion for log link model, ratio 1.14 - very similar to identity link
overdisp_fun(mod0pl)
```

```
##        chisq        ratio          rdf            p 
## 287.42368537   1.13606200 253.00000000   0.06742202
```

```
# Goodness of fit comparisons using the Akaike Information criterion
# See what happens with log link - very little difference - stick with the identity link

cbind(model = c("Normal","Poisson - identity","Poission log link"),AIC = AIC(mod0,mod0p,mod0pl))
```

```
##                     model AIC.df  AIC.AIC
## mod0               Normal     13 1303.833
## mod0p  Poisson - identity     12 1206.082
## mod0pl  Poission log link     12 1207.703
```

For model comparisons a model with an Akaike Information Criterion
(AIC) more than 2 lower than an alternative model is considered a
significant improvement. One that is more than 10 lower would be
considered a substantially better fit (Burnham and Anderson, 2004). As
the focus was on model comparison for this part of the modelling process
maximum likelihood estimation (ML) rather than restricted maximum
likelihood (REML) was used for estimating the model. The Poisson model
(mod0p) with identity link fits much better than the normal model based
on a substantially lower AIC (96). However, the statistical significance
of the model effects (Type III Analysis of Variance Table for the normal
model or the Single term deletions tables for the Poisson models) are
very similar. The Poisson model with log link is a little worse (AIC 2
higher) than the identity link model hence the identity model is
preferred due to the simpler interpretation associated with it. (It
avoids the exponential transformation back to the original scale of
speech lengths required by the log link model with the consequent effect
of creating geometric means and ratios of means on the original scale in
place of differences between the means for significance testing.)

The next step is the removal of the non-significant verse proportion
variable. Only the time period was significant for all 3 models, the LMM
or the 2 GLMMs with Poisson error distribution and identity and log
links respectively.

```
# Removing the non-significant verse group variable - there are some missings in the verse_grp variable so the data sets are different length
mod1<-lmer(mode~ Genre + PlayType2 + f_period3 + (1|Author2),data=plays,REML = F)
#summary(mod1)
anova(mod1)
```

```
## Type III Analysis of Variance Table with Satterthwaite's method
##           Sum Sq Mean Sq NumDF  DenDF F value    Pr(>F)    
## Genre      46.10  11.526     4 269.57  1.6238    0.1685    
## PlayType2  11.93  11.930     1 158.64  1.6807    0.1967    
## f_period3 456.52 228.260     2 217.31 32.1579 5.837e-13 ***
## ---
## Signif. codes:  0 '***' 0.001 '**' 0.01 '*' 0.05 '.' 0.1 ' ' 1
```

```
mod1p<-glmer(mode~ Genre + PlayType2 + f_period3 + (1|Author2),family=poisson(link="identity"),data=plays)
#summary(mod1p)
drop1(mod1p,test="Chisq")
```

```
## Single term deletions
## 
## Model:
## mode ~ Genre + PlayType2 + f_period3 + (1 | Author2)
##           npar    AIC    LRT   Pr(Chi)    
## <none>         1245.4                     
## Genre        4 1245.3  7.894   0.09555 .  
## PlayType2    1 1246.5  3.101   0.07826 .  
## f_period3    2 1297.0 55.642 8.269e-13 ***
## ---
## Signif. codes:  0 '***' 0.001 '**' 0.01 '*' 0.05 '.' 0.1 ' ' 1
```

```
# Check for overdispersion - again slight 
overdisp_fun(mod1p)
```

```
##        chisq        ratio          rdf            p 
## 300.96224827   1.13143702 266.00000000   0.06908131
```

```
mod1pl<-glmer(mode~ Genre + PlayType2 + f_period3 + (1|Author2),family=poisson(link="log"),data=plays)
#summary(mod1pl)
drop1(mod1pl,test="Chisq")
```

```
## Single term deletions
## 
## Model:
## mode ~ Genre + PlayType2 + f_period3 + (1 | Author2)
##           npar    AIC    LRT   Pr(Chi)    
## <none>         1247.1                     
## Genre        4 1246.4  7.325    0.1197    
## PlayType2    1 1247.2  2.152    0.1423    
## f_period3    2 1294.0 50.946 8.654e-12 ***
## ---
## Signif. codes:  0 '***' 0.001 '**' 0.01 '*' 0.05 '.' 0.1 ' ' 1
```

```
overdisp_fun(mod1pl)
```

```
##        chisq        ratio          rdf            p 
## 300.85127933   1.13101985 266.00000000   0.06967235
```

```
cbind(model = c("Normal","Poisson - identity","Poission log link"),AIC = AIC(mod1,mod1p,mod1pl))
```

```
##                     model AIC.df  AIC.AIC
## mod1               Normal     10 1345.388
## mod1p  Poisson - identity      9 1245.365
## mod1pl  Poission log link      9 1247.065
```

Comparing the model using the Poisson distribution for the error term
led to very similar modelling results. The 2 Poisson models were better
by the AIC goodness of fit measure than the normal model.

### Model assumptions - residuals and random effects - Normal

The distribution of residuals for the normal model (S2 Fig 1) are
somewhat right skewed, but overall the distribution appears symmetric
enough that the benefits of the central limit theorem should protect the
reliability of the uncertainties on the means. Notable is one play that
is a very likely a statistical outlier being about 26 higher in mode
than predicted by the model, the next most extreme mode being 10 higher.
Scaling the extreme outlier by the residual SD it was about 10 SD’s
higher than the model prediction (see the Pearson residual plots).
Similar residual distributions are seen for the Poisson identity link
function S2 Fig 2.

S2 Fig 1 Model diagnostics for the model of the mode - Normal
distribution

### Model assumptions - residuals and random effects - Poisson (identity link)

S2 Fig 2 Model diagnostics for the model of the mode - Poisson
distribution - identity link

### Model assumptions - random effects - Normal distribution

S2 Fig 3 Distribution of author random effects for the mode - Normal
distribution.

### Model assumptions - random effects - Poisson (identity link)

S2 Fig 4 Distribution of author random effects for the mode - Poisson
identity link

The assumption of normality of the random effects for the LMM is
approximately met as well in both Poisson model variants.

# Why is the Poisson model a better fit?

### Model coefficients and SEs

The normal and Poisson model coefficients and SEs were compared to
see if there was a clue as to why the Poisson model was a substantially
better fit than the normal model. They are compared in the table below
along with ratios of the difference between the coefficients and
standard errors (Poisson/Normal).

```
##                     coef_norm coef_poisson   SE_norm SE_poisson ratio_coef_p_n ratio_SE_p_n
## (Intercept)         8.0846114    8.0980138 0.4568993  0.4582052      1.0016578    1.0028583
## GenreHistory        1.2609532    1.3458212 0.5810275  0.5787589      1.0673047    0.9960955
## GenreMisc           0.4717702    0.3944019 0.5369417  0.5376990      0.8360043    1.0014104
## GenreTragedy        0.2066754    0.1443835 0.4252063  0.3891793      0.6986003    0.9152718
## GenreTragicomedy   -0.5233433   -0.5353342 0.6298085  0.5100665      1.0229121    0.8098756
## PlayType2Other      0.5014018    0.6220661 0.3867537  0.3539935      1.2406540    0.9152944
## f_period31597-1602 -2.4821087   -2.5251653 0.5347509  0.5423786      1.0173468    1.0142639
## f_period3> 1602    -3.4309690   -3.4828159 0.4292673  0.4409718      1.0151114    1.0272662
```

The significance of fixed effects were similar between the normal
distribution and Poisson identity link models (output above). Some of
the model coefficients did differ by up to 30% but for the important
time variable the normal and Poisson were quite similar varying less
than 2%. The SEs had smaller variations but were generally slightly
lower for the Poisson model, most being less than 10% lower. This did
not seem enough to explain the considerably lower AIC for the Poisson
model, what other explanations could there be?

# Constant variance assumption

As the Poisson model had a better fit (lower AIC by 100 in the new
model runs) this might be due to the variance function that is inherent
in the Poisson distribution, the variance = mean. For the normal
distribution model the variance is assumed to be constant. This effect
was tested by examining the residuals from the models to see if there
was evidence of increasing variability with predicted value in the
normal distribution model residuals. If so then as the Poisson model
would be naturally accounting for this would be expected to be a better
fit.

Following are 4 regression outputs matching the 4 plots of the
absolute residuals against predicted values in S2 Fig 5. Note that the
author random effects are part of the predicted values. The fitted
regressions lines provide an estimate of the relationship between the SD
of the residuals and predicted value, Davidian and Carroll (1987).

```
## [1] "Normal SD function - unscaled"
## 
## Call:
## lm(formula = abs_resid ~ pred)
## 
## Residuals:
##     Min      1Q  Median      3Q     Max 
## -2.0670 -0.9269 -0.4489  0.3410 25.8483 
## 
## Coefficients:
##             Estimate Std. Error t value Pr(>|t|)   
## (Intercept)  0.25405    0.47208   0.538  0.59091   
## pred         0.20161    0.07273   2.772  0.00595 **
## ---
## Signif. codes:  0 '***' 0.001 '**' 0.01 '*' 0.05 '.' 0.1 ' ' 1
## 
## Residual standard error: 2.141 on 273 degrees of freedom
## Multiple R-squared:  0.02738,    Adjusted R-squared:  0.02382 
## F-statistic: 7.685 on 1 and 273 DF,  p-value: 0.005953
```

```
## [1] "Normal SD function - Pearson"
## 
## Call:
## lm(formula = abs_resid ~ pred)
## 
## Residuals:
##     Min      1Q  Median      3Q     Max 
## -0.7758 -0.3479 -0.1685  0.1280  9.7020 
## 
## Coefficients:
##             Estimate Std. Error t value Pr(>|t|)   
## (Intercept)  0.09536    0.17719   0.538  0.59091   
## pred         0.07567    0.02730   2.772  0.00595 **
## ---
## Signif. codes:  0 '***' 0.001 '**' 0.01 '*' 0.05 '.' 0.1 ' ' 1
## 
## Residual standard error: 0.8035 on 273 degrees of freedom
## Multiple R-squared:  0.02738,    Adjusted R-squared:  0.02382 
## F-statistic: 7.685 on 1 and 273 DF,  p-value: 0.005953
```

```
## [1] "Poisson SD function - unscaled"
## 
## Call:
## lm(formula = abs_resid ~ pred)
## 
## Residuals:
##     Min      1Q  Median      3Q     Max 
## -2.0848 -0.9083 -0.5022  0.3729 25.8053 
## 
## Coefficients:
##             Estimate Std. Error t value Pr(>|t|)   
## (Intercept)  0.16798    0.46622   0.360  0.71889   
## pred         0.21598    0.07189   3.004  0.00291 **
## ---
## Signif. codes:  0 '***' 0.001 '**' 0.01 '*' 0.05 '.' 0.1 ' ' 1
## 
## Residual standard error: 2.143 on 273 degrees of freedom
## Multiple R-squared:  0.03201,    Adjusted R-squared:  0.02846 
## F-statistic: 9.027 on 1 and 273 DF,  p-value: 0.002908
```

```
## [1] "Poisson SD function - Pearson"
## 
## Call:
## lm(formula = abs_resid ~ pred)
## 
## Residuals:
##     Min      1Q  Median      3Q     Max 
## -0.7023 -0.3486 -0.2140  0.1659 10.7458 
## 
## Coefficients:
##             Estimate Std. Error t value Pr(>|t|)  
## (Intercept)  0.30781    0.18638   1.652   0.0998 .
## pred         0.04677    0.02874   1.627   0.1048  
## ---
## Signif. codes:  0 '***' 0.001 '**' 0.01 '*' 0.05 '.' 0.1 ' ' 1
## 
## Residual standard error: 0.8565 on 273 degrees of freedom
## Multiple R-squared:  0.009607,   Adjusted R-squared:  0.00598 
## F-statistic: 2.648 on 1 and 273 DF,  p-value: 0.1048
```

S2 Fig 5 Model diagnostics - variance function comparison between the
normal and Poisson (identity link) models

All residual plots in S2 Fig 5 (the extreme outlier not shown to
improve axis scaling) show a positive relationship between the absolute
residuals on the y axis against predicted value on the x axis. The raw
or unscaled absolute residuals for the normal distribution model in the
top left plot show a slight positive relationship with predicted value.
This is confirmed by a significant regression slope in the first of the
4 regression model outputs, B(SE) = 0.202(0.073), p = .006. The
regression line defines the standard deviation relationships between
residuals and predicted value, for a 1 unit increase in the predicted
mean of the mode the SD of residuals will increase by 0.202.

For the normal model distribution plot at top right where Pearson
residuals have been used to determine the absolute residuals, the
unscaled residuals have been scaled using the residual standard
deviation from the normal model distribution (a constant, for this model
being 2.66, output not shown). Therefore it is not surprising that the
regression line for the normal distribution Pearson residuals has
exactly the same significance, p=.006, and the slope of the regression
line 0.0757 is equal to the regression slope for the unscaled absolute
residuals divided by the residual SD, i.e. 0.2016/2.266.

For the Poisson distribution model there is a similar relationship
between the unscaled and Pearson (scaled) residuals with respect to
predicted values. The lower left plot with the Poisson unscaled absolute
residuals is somewhat similar to the unscaled residuals from the normal
distribution unscaled residuals. This is confirmed with the Poisson
regression line having very similar slope, SE and significance,
0.216(0.072), p=.003, to that of the normal absolute unscaled residual
regression.

The important difference is found in the final Poisson plot (lower
right) with the absolute Pearson (scaled) residuals. The relationship is
much flatter with the regression slope about 75% smaller,
B(SE)=0.0468(0.029), and not significant with p=.10. This smaller slope
indicates the variance function inherent in the Poisson distribution has
removed a portion of the variability relationship in the residuals. The
Poisson variability function being SD = predicted value1/2.
That is smaller predicted means for the mode have lower variability than
those with higher mean mode.

If this is correct and is the main reason for the better fit (lower
AIC) of the Poisson model then the normal distribution model could be
adapted with a variance function based on the same equation to be
equivalent to the Poisson model with identity link. This table compares
the goodness of fit of the 4 models.

```
##                                         model AIC.df  AIC.AIC
## mod1                                   Normal     10 1345.388
## mod1v            Normal with Poisson variance     10 1329.630
## mod1v2 Normal with residual variance function     10 1326.115
## mod1p                      Poisson - identity      9 1245.365
```

Adding a variance function to the normal model did improve the fit,
but not enough to explain the much better fit of the Poisson model
compared to the normal distribution based model. Using a variance
function based on the Poisson distribution improved the model fit with a
reduction in the AIC of 16. Choosing a variance function based on a
regression of the normal model absolute residuals improved it a little
more, AIC reduced by only 3 more than the model with the Poisson
variance function. These changes were still a long way short of the AIC
change of 100 when comparing the Poisson model to the base normal model
(Without weights based on variance functions).

Is there any other explanation for this much better fit (i.e. lower
AIC by 100) of the Poisson model to the data?

# What about equality of random effects

S2 Fig 6 Random effects for the normal distribution model

S2 Fig 7 Random effects for the Poisson distributoin model with identity
link function

S2 Fig 8 Random effect comparison between the normal and Poisson
(identity link) models

The random effect comparison showed there were some differences but
are these did not seem large enough to explain the difference in model
goodness of fit with the Poisson having lower AIC by 100.

### Check effect of outlying mode of 33

Are the normal and Poisson models that different in AIC once the very
discordant play is removed?

```
# Refit model with mode=33 removed

# Normal model
mod1a<-lmer(mode~ Genre + PlayType2 + f_period3 + (1|Author2),data=plays3,REML=F)
# summary(mod1a)
# anova(mod1a)

# Poisson distribution model
mod1ap<-glmer(mode~ Genre + PlayType2 + f_period3 + (1|Author2),family=poisson(link="identity"),data=plays3)
# summary(mod1ap)
# drop1(mod1ap,test="Chisq")

# Check for overdispersion - now the Poisson is quite underdispersed with the omission of a single observation - this is an important clue
overdisp_fun(mod1ap)
```

```
##       chisq       ratio         rdf           p 
## 185.0162243   0.6981744 265.0000000   0.9999461
```

```
# AIC with the outlying observation removed

# Compare models
cbind(model = c("Normal - outlier removed","Poisson - identity outlier removed"),AIC = AIC(mod1a,mod1ap))
```

```
##                                     model AIC.df  AIC.AIC
## mod1a            Normal - outlier removed     10 1202.604
## mod1ap Poisson - identity outlier removed      9 1174.226
```

A large part of the AIC difference between the normal and Poisson
models was reduced when the outlying mode of 33 was removed. The AIC
difference of 100 with the outlying observation included was reduced to
28 when it was removed. Surprisingly the one play with a very large mode
was affecting the difference in model results between normal and Poisson
substantially. Notable also is the overdispersion check on the Poisson
model, now the ratio is substantially lower than 1 at 0.70. This is
interpreted as underdispersion, meaning the variation of the residuals
is now substantially less than what would be expected from the Poisson
distribution. The interpretation of this will be apparent from an
analysis that follows.

# Model diagnostics for the model with the play with mode 33 removed

The plots for both the normal and Poisson identity link model are not
shown but the residual patterns are largely unchanged from the residual
plots above with the difference that the large outlier is now gone.

Variability estimates - Normal model with all data

```
...
## 
## Random effects:
##  Groups   Name        Variance Std.Dev.
##  Author2  (Intercept) 0.1885   0.4342  
##  Residual             7.0981   2.6642  
## Number of obs: 275, groups:  Author2, 32
## 
...
```

Variability estimates Normal model outlier play with mode 33
removed

```
...
## 
## Random effects:
##  Groups   Name        Variance Std.Dev.
##  Author2  (Intercept) 0.4353   0.6598  
##  Residual             4.1136   2.0282  
## Number of obs: 274, groups:  Author2, 32
## 
...
```

Comparing the author random effect estimates for the normal models
with and without the play with mode 33 shows that the distribution of
author differences increased from SD 0.43 to 0.66. This indicates a
somewhat stronger author effect on the mode of speech length with
greater differences between authors.

### Constant variance assumption - mode 33 removed

Repeating the variance function analysis above with the outlier with
mode 33 removed and comparing the normal and Poisson models again.

```
## [1] "Normal SD function - unscaled"
## 
## Call:
## lm(formula = abs_resid ~ pred)
## 
## Residuals:
##     Min      1Q  Median      3Q     Max 
## -2.1425 -0.7137 -0.3729  0.2866  9.2654 
## 
## Coefficients:
##             Estimate Std. Error t value Pr(>|t|)    
## (Intercept) -0.11213    0.29430  -0.381    0.704    
## pred         0.23307    0.04581   5.088 6.76e-07 ***
## ---
## Signif. codes:  0 '***' 0.001 '**' 0.01 '*' 0.05 '.' 0.1 ' ' 1
## 
## Residual standard error: 1.419 on 272 degrees of freedom
## Multiple R-squared:  0.0869, Adjusted R-squared:  0.08354 
## F-statistic: 25.89 on 1 and 272 DF,  p-value: 6.759e-07
```

```
## [1] "Normal SD function - Pearson"
## 
## Call:
## lm(formula = abs_resid ~ pred)
## 
## Residuals:
##     Min      1Q  Median      3Q     Max 
## -1.0564 -0.3519 -0.1838  0.1413  4.5683 
## 
## Coefficients:
##             Estimate Std. Error t value Pr(>|t|)    
## (Intercept) -0.05528    0.14510  -0.381    0.704    
## pred         0.11492    0.02259   5.088 6.76e-07 ***
## ---
## Signif. codes:  0 '***' 0.001 '**' 0.01 '*' 0.05 '.' 0.1 ' ' 1
## 
## Residual standard error: 0.6995 on 272 degrees of freedom
## Multiple R-squared:  0.0869, Adjusted R-squared:  0.08354 
## F-statistic: 25.89 on 1 and 272 DF,  p-value: 6.759e-07
```

```
## [1] "Poisson SD function - unscaled"
## 
## Call:
## lm(formula = abs_resid ~ pred)
## 
## Residuals:
##     Min      1Q  Median      3Q     Max 
## -1.9724 -0.8619 -0.4007  0.4263 10.1484 
## 
## Coefficients:
##             Estimate Std. Error t value Pr(>|t|)    
## (Intercept)  0.04497    0.32028   0.140    0.888    
## pred         0.22258    0.04992   4.459 1.21e-05 ***
## ---
## Signif. codes:  0 '***' 0.001 '**' 0.01 '*' 0.05 '.' 0.1 ' ' 1
## 
## Residual standard error: 1.523 on 272 degrees of freedom
## Multiple R-squared:  0.06812,    Adjusted R-squared:  0.06469 
## F-statistic: 19.88 on 1 and 272 DF,  p-value: 1.206e-05
```

```
## [1] "Poisson SD function - Pearson"
## 
## Call:
## lm(formula = abs_resid ~ pred)
## 
## Residuals:
##     Min      1Q  Median      3Q     Max 
## -0.6685 -0.3648 -0.1768  0.1648  4.6650 
## 
## Coefficients:
##             Estimate Std. Error t value Pr(>|t|)  
## (Intercept)  0.27496    0.12511   2.198   0.0288 *
## pred         0.04681    0.01950   2.401   0.0170 *
## ---
## Signif. codes:  0 '***' 0.001 '**' 0.01 '*' 0.05 '.' 0.1 ' ' 1
## 
## Residual standard error: 0.5948 on 272 degrees of freedom
## Multiple R-squared:  0.02075,    Adjusted R-squared:  0.01715 
## F-statistic: 5.763 on 1 and 272 DF,  p-value: 0.01704
```

S2 Fig 9 Model diagnostics with play with mode 33 removed - SD function
comparison between the normal and Poisson (identity link) models.

With the removal of the outlier play with mode = 33 the variance
functions for residuals were similar compared to the SD functions fitted
above with the outlier play included. All residual plots in S2 Fig 9
again show the relationship between the absolute residuals and predicted
value. The SD function for the unscaled residual for normal distribution
model above using all data was B(SE) = 0.215(0.074), p = .004, with the
outlier removed this relationship was a little stronger was
0.233(0.049), p <.001. As before the variance adjusted Poisson
Pearson absolute residuals (lower right plot) the relationship is still
flatter with the regression slope about 80% smaller, B(SE)=0.047(0.020),
and now significant, p=.02. This reduction in slope again confirms that
the benefit of the inherent Poisson variance function, however the
remaining positive slope suggests that the variability of the model
residuals is a little greater than that built into the Poisson
model.

# Compare normal model with weighting function to Poisson - outlier mode 33 removed

Now that the outlier impact has been removed how well does the normal
distribution model (adjusted with weights to account for the
non-constant variance) compare with the Poisson distribution model?

```
# Add the weighting function equivalent to the Poisson variance, for a Poisson process with mean N, var= N
# Optimal weights are 1/var
plays3$poisson_weights3 = 1/predict(mod1a)

# Fit the normal model with Poisson weights 
mod1avp<-lmer(mode~ Genre + PlayType2 + f_period3 + (1|Author2),weight = poisson_weights3,data=plays3,REML=F)
# summary(mod1avp)
# anova(mod1avp)

# Fit the normal model with weights based on the normal distribution model absolute residuals
plays3$normal_weights3 = 1/((-0.112 + 0.2331*predict(mod1a))^2)
mod1avn<-lmer(mode~ Genre + PlayType2 + f_period3 + (1|Author2),weight = normal_weights3,data=plays3,REML=F)
# summary(mod1avn)
# anova(mod1avn)

# Now the normal distribution with weighting is a better fit than the Poisson
cbind(model = c("Normal no SD function","Poisson","Normal with Poisson SD function","Normal with normal SD function"),AIC = AIC(mod1a,mod1ap,mod1avp,mod1avn))
```

```
##                                   model AIC.df  AIC.AIC
## mod1a             Normal no SD function     10 1202.604
## mod1ap                          Poisson      9 1174.226
## mod1avp Normal with Poisson SD function     10 1164.666
## mod1avn  Normal with normal SD function     10 1143.405
```

The analysis above shows that with the outlier play removed the
normal distribution model without any weights (AIC 1203) is not as good
as the Poisson model (AIC 1177), with the Poisson model having an AIC 28
lower than that of the normal model. Adding weights to the the normal
distribution model that were based on the Poisson distribution led to
normal model a better fit than the equivalent Poisson (AIC 10
lower).

The biggest improvement was obtained by using an SD function derived
from the unscaled normal model residuals leading to an even better fit
with an AIC of 1143 being 31 lower than that of the Poisson identity
link model. As models with AIC lower by more than 10 are considered
indicative of a substantially better fit (Burnham and Anderson 2004),
this better fit indicates the variability function used in the normal
model is more appropriate for this data set than the one based on the
Poisson model. Why this is so in examined below.

S2 Fig 10 Comparison of SD functions with the outlier play (mode 33)
removed. The SD function is based on the normal distribution model. The
Poisson SD function is calculated as predicted value1/2 from
the normal model predicted values

```
##          min1    mean1     max1
## [1,] 1.408263 1.959847 2.483559
```

The nature of the difference in the residual variability functions is
shown in S2 Fig 10 above The lower plot in black and fitted line is the
SD function obtained from normal distribution residuals (SD = -0.112 +
0.2331.Predicted). The upper set of points show the SD function
calculated based on the Poisson model using the normal model (without
weighting) predicted values, SD(Poisson) = predicted1/2. They
both show a similar pattern with the variability (SD) increasing with
predicted value. The red line for the Poisson SD function shows how this
function behaves over an extended range (beyond the range of the data in
this study). Noteworthy is the substantial offset with the Poisson
distribution based function being about 1 SD higher than those based on
the normal model residuals. Comparing the two SD functions over the
range of the data shows that on average the Poisson SD function is about
2.0 times larger than the normal distribution SD function. This fits
well with the earlier observation about the underdispersion of the
Poisson model because the variability of residuals is less than what
would be expected based on the Poisson distribution.

# Carry out iteration to obtain the best estimate of the SD function for weighting

Ideally the SD function for the normal model would be refined by
refitting the model multiple times until the model estimates and SD
function parameters converge. This was done but convergence could not be
obtained. The plot of AIC in S2 Fig 11 shows an oscillating behaviour
from about iteration 4. The AIC estimates from the oscillations were all
substantially lower than the model without weights based on an SD
function. The tables below the figure of parameter estimates and SD
function parameters from the iteration process showed only small changes
in parameter estimates for the fixed effect parameters and the SD
function parameters.

```
options(width=110,digits=5)
# Test the fit to the SD function with 3 replicates for each random effect
form1 <- mode~ Genre + PlayType2 + f_period3 + (1|Author2)
iter20 = lmm_sd_function(plays3,form1,k=20)


# Convergence not attained - looks like oscillating behaviour from cycle 4 onward and stabilised oscillation 
# from iteration 7 onwards

op=par(mfrow=c(3,1))
  plot(iter20$est[,"AIC"]~iter20$est[,"iteration"],type="b",main="iterative scheme parameters, AIC & SD function slope and intercept",ylab="AIC", xlab="Iteration number")
  text(2,1200,"AIC for Iteration 0 is from the model without weights",adj=0)

  plot(iter20$est[,"sd_slope"]~iter20$est[,"iteration"],type="b",ylab="SD slope", xlab="Iteration number")
  text(-0.5,0.27,"SD slope for Iteration 0 is from the first model without weights",adj=0)
  
  plot(iter20$est[,"sd_intercept"]~iter20$est[,"iteration"],type="b",ylab="SD intercept", xlab="Iteration number")
  text(-0.5,-0.4,"SD intercept for Iteration 0 is from the first model without weights",adj=0)
```

S2 Fig 11 AIC results for an iterative fitting process to refine the SD
fuction

```
par(op)

# # Model fit and fixed effect parameters - genre & play type
# iter20$est[,1:7]
# 
# # Model fit and fixed effect parameters - time period
# iter20$est[,8:10]
# 
# # Model fit and SD function parameters
# iter20$est[,c(1:2,11:12)]
# 
# # Examine the SD function for the 2 oscillating clusters
# # Lower AIC cluster ~ 1110
# iter20$est[c(1,3,5,7,9,11,13,15,17,19,21),c(1:2,11:12)]
# 
# # Higher AIC cluster ~ 1170
# iter20$est[c(1,3,5,7,9,11,13,15,17,19)+1,c(1:2,11:12)]
```

This was exemplified in S2 Fig 12 where the SD functions from
iterations 9 to 20 where the oscillating behaviour had stabilised are
compared to the SD function from the first iteration. The differences
were not large.

S2 Fig 12 Comparison of SD functions from models at steps 1 and 9 to 20
in the iterative scheme

Plots of absolute residuals (unscaled and Pearson) and predicted
values from models from the first, 19th and 20th iterations are compared
in S2 Fig 13 along with the fitted SD functions. These last 2 iterations
being representative of the oscillating behaviour in the AIC goodness of
fit measure. The plots appear similar except for one unusual feature, at
iteration 19, a single predicted value is much larger at about 12.5. All
other predictions were in the range a little lower than 4 to 10. This
was the iteration where the model had the poorest goodness of fit with
an AIC of about 1163 and then on the very next iteration the quality of
the fit was the best observed with AIC dropping to about 1107.

```
# Save the model from the second last iteration above by running one less iteration
form1 <- mode~ Genre + PlayType2 + f_period3 + (1|Author2)
iter19 = lmm_sd_function(plays3,form1,k=19)

# Compare models
AIC(mod1avn,iter19$model,iter20$model)
```

```
##              df    AIC
## mod1avn      10 1143.4
## iter19$model 10 1163.4
## iter20$model 10 1107.1
```

```
# Compare SD functions
op=par(mfrow=c(3,2))
  sd_resid(mod1avn,"Iteration 1 AIC 1155")
```

```
## [1] "Iteration 1 AIC 1155 SD function - unscaled"
## 
## Call:
## lm(formula = abs_resid ~ pred)
## 
## Residuals:
##    Min     1Q Median     3Q    Max 
## -2.214 -0.774 -0.383  0.361  9.288 
## 
## Coefficients:
##             Estimate Std. Error t value Pr(>|t|)    
## (Intercept)  -0.1444     0.3058   -0.47     0.64    
## pred          0.2400     0.0481    4.99  1.1e-06 ***
## ---
## Signif. codes:  0 '***' 0.001 '**' 0.01 '*' 0.05 '.' 0.1 ' ' 1
## 
## Residual standard error: 1.47 on 272 degrees of freedom
## Multiple R-squared:  0.0838, Adjusted R-squared:  0.0804 
## F-statistic: 24.9 on 1 and 272 DF,  p-value: 1.1e-06
```

```
## [1] "Iteration 1 AIC 1155 SD function - Pearson"
## 
## Call:
## lm(formula = abs_resid ~ pred)
## 
## Residuals:
##    Min     1Q Median     3Q    Max 
## -0.739 -0.430 -0.237  0.182  5.432 
## 
## Coefficients:
##             Estimate Std. Error t value Pr(>|t|)    
## (Intercept)   0.5407     0.1485    3.64  0.00032 ***
## pred          0.0214     0.0234    0.92  0.36051    
## ---
## Signif. codes:  0 '***' 0.001 '**' 0.01 '*' 0.05 '.' 0.1 ' ' 1
## 
## Residual standard error: 0.715 on 272 degrees of freedom
## Multiple R-squared:  0.00307,    Adjusted R-squared:  -0.00059 
## F-statistic: 0.839 on 1 and 272 DF,  p-value: 0.361
```

```
  sd_resid(iter19$model,"Iteration 19 AIC 1170")
```

```
## [1] "Iteration 19 AIC 1170 SD function - unscaled"
## 
## Call:
## lm(formula = abs_resid ~ pred)
## 
## Residuals:
##    Min     1Q Median     3Q    Max 
## -2.156 -0.640 -0.349  0.360  6.537 
## 
## Coefficients:
##             Estimate Std. Error t value Pr(>|t|)    
## (Intercept)  -0.4451     0.2602   -1.71    0.088 .  
## pred          0.2819     0.0403    6.99  2.2e-11 ***
## ---
## Signif. codes:  0 '***' 0.001 '**' 0.01 '*' 0.05 '.' 0.1 ' ' 1
## 
## Residual standard error: 1.32 on 272 degrees of freedom
## Multiple R-squared:  0.152,  Adjusted R-squared:  0.149 
## F-statistic: 48.8 on 1 and 272 DF,  p-value: 2.17e-11
```

```
## [1] "Iteration 19 AIC 1170 SD function - Pearson"
## 
## Call:
## lm(formula = abs_resid ~ pred)
## 
## Residuals:
##    Min     1Q Median     3Q    Max 
## -0.818 -0.423 -0.199  0.253  4.247 
## 
## Coefficients:
##             Estimate Std. Error t value Pr(>|t|)   
## (Intercept)   0.3159     0.1349    2.34   0.0199 * 
## pred          0.0573     0.0209    2.74   0.0065 **
## ---
## Signif. codes:  0 '***' 0.001 '**' 0.01 '*' 0.05 '.' 0.1 ' ' 1
## 
## Residual standard error: 0.682 on 272 degrees of freedom
## Multiple R-squared:  0.0269, Adjusted R-squared:  0.0233 
## F-statistic: 7.51 on 1 and 272 DF,  p-value: 0.00653
```

```
  sd_resid(iter20$model,"Iteration 20 AIC 1110")
```

```
## [1] "Iteration 20 AIC 1110 SD function - unscaled"
## 
## Call:
## lm(formula = abs_resid ~ pred)
## 
## Residuals:
##    Min     1Q Median     3Q    Max 
## -2.037 -0.791 -0.429  0.342 10.759 
## 
## Coefficients:
##             Estimate Std. Error t value Pr(>|t|)    
## (Intercept)  -0.0429     0.3180   -0.13     0.89    
## pred          0.2291     0.0508    4.51  9.8e-06 ***
## ---
## Signif. codes:  0 '***' 0.001 '**' 0.01 '*' 0.05 '.' 0.1 ' ' 1
## 
## Residual standard error: 1.55 on 272 degrees of freedom
## Multiple R-squared:  0.0695, Adjusted R-squared:  0.0661 
## F-statistic: 20.3 on 1 and 272 DF,  p-value: 9.79e-06
```

```
## [1] "Iteration 20 AIC 1110 SD function - Pearson"
## 
## Call:
## lm(formula = abs_resid ~ pred)
## 
## Residuals:
##    Min     1Q Median     3Q    Max 
## -0.681 -0.482 -0.207  0.233  3.187 
## 
## Coefficients:
##             Estimate Std. Error t value Pr(>|t|)    
## (Intercept)  0.70736    0.14530    4.87  1.9e-06 ***
## pred        -0.00285    0.02323   -0.12      0.9    
## ---
## Signif. codes:  0 '***' 0.001 '**' 0.01 '*' 0.05 '.' 0.1 ' ' 1
## 
## Residual standard error: 0.709 on 272 degrees of freedom
## Multiple R-squared:  5.55e-05,   Adjusted R-squared:  -0.00362 
## F-statistic: 0.0151 on 1 and 272 DF,  p-value: 0.902
```

S2 Fig 13 Comparison of absolute residual plots on which the SD
functions were based for models at steps 1 and 19 and 20 in the
iterative scheme

```
par(op)
```

After further investigation it was determined this high predicted
value was due to one of the authors (Daniel Samuel). The random effect
estimate (BLUP - best linear unbiased prediction) for the mean of his
plays changed becoming much larger at iteration 19 when compared to the
rest of the authors. However on the next iteration this became smaller
and was within the range of all other authors. See the middle plot in S2
Fig 14 where the estimate for Daniel Samuel becomes much higher (about
4.5) compared to the range of other authors (about -1.5 to +1.0). At the
following iteration (20) the range of the BLUP predictions shrinks
greatly to be in the range about -0.3 to +0.3 and Daniel Samuel’s
estimate is now about 0.1. Comparing the BLUPs for iterations 19 and 20
with the first iteration where the random effects BLUPs range from about
-0.5 to +0.5 with Daniel Samuel’s being a little higher at about +0.90.
This suggests a problem in model fitting due to this author.

S2 Fig 14 Comparison of random effects from models at steps 1 and 19 and
20 in the iterative scheme

To further understand this the SD functions from the 19th and 20th
iterations which gave quite different AIC values were used in place of
the SD function used at the first iteration to see what impact they
would have on the goodness of fit.

The table below summarises (again) the AIC goodness of fit for the 4
important models in this study. To this now are added two additional
models where the SD function from the first iteration was replaced by
the SD functions from the 19th and 20th iterations. Would they lead to
the large divergence in AIC as was observed in the iteration scheme
output? The answer was no, the AICs were quite similar to that of the
normal model from the first iteration. This further supports that the
apparent improvement of the model fit at iteration 20 was illusory,
rather it was based on the weights used in the 20th iteration being
calculated from the predicted values from the previous iteration (the
19th) giving the illusion of a better fit. However when the SD function
for the 20th iteration was used the AIC was no different to that from
the SD function for the first iteration.

```
##                                                      model AIC.df AIC.AIC
## mod1a                                Normal no SD function     10  1202.6
## mod1ap                          Poisson with identity link      9  1174.2
## mod1avp                    Normal with Poisson SD function     10  1164.7
## mod1avn      Normal - SD function from the first iteration     10  1143.4
## mod1a_iter19  Normal - SD function from the 19th iteration     10  1139.3
## mod1a_iter20  Normal - SD function from the 20th iteration     10  1144.6
```

Examining the residual plots for the model from the first iteration
with an SD function used for weighting revealed a single play with a
very high Pearson residual, about +9SDs, S2 Fig 15.

S2 Fig 15 Model diagnostics for the model from the first iteration

Investigation of the high Pearson residual showed it was one of
Daniel Samuel’s 2 plays, Philotas.

```
# Identify plays with higher residual modes that don't fit the model so well
plays3$pearson_resid = residuals(mod1a_iter20,type="pearson", scaled=FALSE)
plays_high_resid = arrange(filter(plays3,pearson_resid >= 2),desc(pearson_resid))
print(plays_high_resid[,c(14,1,7,10,24,50)])
```

```
##    Date                            TextTitle                Author   Genre mode pearson_resid
## 1  1604                             Philotas         Daniel Samuel Tragedy   16        8.4861
## 2  1633                        Tale of a Tub            Jonson Ben  Comedy    9        4.7158
## 3  1590          True Chronicle of King Leir             Uncertain    Misc   18        4.2154
## 4  1638                   Unfortunate Lovers      Davenant William Tragedy    9        4.1971
## 5  1624                        Game at Chess      Middleton Thomas    Misc   10        4.1841
## 6  1589                    Battle of Alcazar          Peele George    Misc   16        3.5041
## 7  1594     Love of David and Fair Bathsheba          Peele George    Misc   16        3.5041
## 8  1608                  Humor Out of Breath              Day John  Comedy    9        3.4218
## 9  1562                    Ferrex and Porrex      Sackville Thomas Tragedy   15        3.3888
## 10 1608                          Dumb Knight    Markham and Machin  Comedy    9        3.2991
## 11 1587              1 Tamburlaine the Great   Marlowe Christopher    Misc   15        2.8535
## 12 1588              2 Tamburlaine the Great   Marlowe Christopher    Misc   15        2.8535
## 13 1610                Christian Turned Turk        Daborne Robert Tragedy    8        2.8462
## 14 1607                   Insatiate Countess Marston and Barkstead Tragedy    8        2.8462
## 15 1604 1 If You Know Not Me You Know Nobody        Heywood Thomas History    9        2.6323
## 16 1605 2 If You Know Not Me You Know Nobody        Heywood Thomas History    9        2.6323
## 17 1591     2 Troublesome Reign of King John             Uncertain History   16        2.6134
## 18 1597                 Humorous Day's Mirth        Chapman George  Comedy    9        2.5810
## 19 1599                       Old Fortunatus         Dekker Thomas  Comedy    9        2.5442
## 20 1630                             City Wit         Brome Richard  Comedy    8        2.3629
## 21 1602                      Gentleman Usher        Chapman George  Comedy    9        2.2898
## 22 1601                         Satiromastix         Dekker Thomas  Comedy    9        2.2549
## 23 1599               Warning for Fair Women             Uncertain Tragedy    9        2.0903
## 24 1598              Englishmen for My Money      Haughton William  Comedy    9        2.0315
```

```
# Daniel Samuel's plays
plays_ds = arrange(filter(plays3,Author == "Daniel Samuel"),desc(pearson_resid))
print(plays_ds[,c(14,1,7,10,24,50)])
```

```
##   Date TextTitle        Author   Genre mode pearson_resid
## 1 1604  Philotas Daniel Samuel Tragedy   16        8.4861
## 2 1593 Cleopatra Daniel Samuel Tragedy    7       -1.0617
```

```
# Drop Pilotas and see if the iteration scheme converges
plays4 = filter(plays3,TextTitle != "Philotas")

# As Daniel Samuel now has only 1 play left his remaining play should be assigned to the Single play Author group
subset(plays4, Author == "Daniel Samuel")[,c(14,1,7,10,24,45,50)]
```

```
##    Date TextTitle        Author   Genre mode       Author2 pearson_resid
## 59 1593 Cleopatra Daniel Samuel Tragedy    7 Daniel Samuel       -1.0617
```

```
plays4$Author2[59] = "Single Play Author"
subset(plays4, Author == "Daniel Samuel")[,c(14,1,7,10,24,45,50)]
```

```
##    Date TextTitle        Author   Genre mode            Author2 pearson_resid
## 59 1593 Cleopatra Daniel Samuel Tragedy    7 Single Play Author       -1.0617
```

After removing Daniel Samuel’s Philotas from the analysis and moving
the remaining play Cleopatra to the single author group the iteration
scheme converged, S2 Fig 16 and details below.

S2 Fig 16 AIC results for a repeat of the iterative fitting process to
refine the SD fuction after dropping the play Philotas with an extreme
Pearson residual of 9 that appeared to be the reason for the oscillatory
behaviour of the previous run of the iterative scheme

```
## [1] "SD function parameters"
```

```
##       iteration    AIC sd_intercept sd_slope
##  [1,]         0 1167.7      0.00000  0.00000
##  [2,]         1 1094.2     -0.19290  0.23954
##  [3,]         2 1096.1     -0.19665  0.24471
##  [4,]         3 1095.6     -0.20480  0.24562
##  [5,]         4 1095.7     -0.20512  0.24577
##  [6,]         5 1095.7     -0.20549  0.24582
##  [7,]         6 1095.7     -0.20549  0.24582
##  [8,]         7 1095.7     -0.20550  0.24583
##  [9,]         8 1095.7     -0.20550  0.24582
## [10,]         9 1095.7     -0.20550  0.24582
## [11,]        10 1095.7     -0.20550  0.24582
## [12,]        11 1095.7     -0.20550  0.24582
## [13,]        12 1095.7     -0.20550  0.24582
## [14,]        13 1095.7     -0.20550  0.24582
## [15,]        14 1095.7     -0.20550  0.24582
## [16,]        15 1095.7     -0.20550  0.24582
## [17,]        16 1095.7     -0.20550  0.24582
## [18,]        17 1095.7     -0.20550  0.24582
## [19,]        18 1095.7     -0.20550  0.24582
## [20,]        19 1095.7     -0.20550  0.24582
## [21,]        20 1095.7     -0.20550  0.24582
```

From this investigation it was concluded that dropping the play
Philotas was necessary to reliably determine the SD function, the
intercept and slope from this fitting scheme to be used for the the
final normal distribution model, SD function = -0.2055 +
0.2458\*predicted.

### Final model for the mode

Following the investigation above it was concluded that a model for
the mode based on excluding the outlier play with mode 33 and fitting a
LMM (i.e. using the normal distribution to model the residuals) with
model weights based on a SD function to adjust for non constant
variability was a better choice than one based on the Poisson
distribution. This final model has been used in the Supplementary 1 file
for the analysis of the mode. Stable estimates of the SD function
however could only be obtained after excluding the play Philotas.

The initial and final normal distribution models with full output are
repeated here for ease of reference and comparison to the Poisson
model.

```
# Normal model
mod1a_final<-lmer(mode~ Genre + PlayType2 + f_period3 + (1|Author2),data=plays4,REML=F)
summary(mod1a_final)
```

```
## Linear mixed model fit by maximum likelihood . t-tests use Satterthwaite's method ['lmerModLmerTest']
## Formula: mode ~ Genre + PlayType2 + f_period3 + (1 | Author2)
##    Data: plays4
## 
##      AIC      BIC   logLik deviance df.resid 
##   1167.7   1203.8   -573.9   1147.7      263 
## 
## Scaled residuals: 
##    Min     1Q Median     3Q    Max 
## -3.980 -0.518 -0.159  0.333  4.411 
## 
## Random effects:
##  Groups   Name        Variance Std.Dev.
##  Author2  (Intercept) 0.326    0.571   
##  Residual             3.708    1.926   
## Number of obs: 273, groups:  Author2, 31
## 
## Fixed effects:
##                    Estimate Std. Error       df t value Pr(>|t|)    
## (Intercept)          8.4669     0.3557 130.2925   23.80  < 2e-16 ***
## GenreHistory         0.9180     0.4309 269.8577    2.13    0.034 *  
## GenreMisc            0.3324     0.3948 272.6412    0.84    0.401    
## GenreTragedy        -0.4243     0.3179 272.4058   -1.33    0.183    
## GenreTragicomedy    -0.5318     0.4647 272.9636   -1.14    0.253    
## PlayType2Other      -0.0211     0.2999 185.4534   -0.07    0.944    
## f_period31597-1602  -2.5038     0.3985 258.2481   -6.28  1.4e-09 ***
## f_period3> 1602     -3.7243     0.3300 177.9427  -11.29  < 2e-16 ***
## ---
## Signif. codes:  0 '***' 0.001 '**' 0.01 '*' 0.05 '.' 0.1 ' ' 1
## 
## Correlation of Fixed Effects:
##             (Intr) GnrHst GnrMsc GnrTrgd GnrTrgc PlyT2O f_3159
## GenreHistry -0.407                                            
## GenreMisc   -0.491  0.288                                     
## GenreTragdy -0.344  0.287  0.302                              
## GenrTrgcmdy -0.210  0.193  0.184  0.286                       
## PlyTyp2Othr -0.347  0.224  0.023  0.119   0.111               
## f_31597-160 -0.494 -0.050  0.266  0.058   0.010  -0.004       
## f_prd3>1602 -0.681  0.160  0.270 -0.038  -0.087   0.063  0.515
```

```
anova(mod1a_final)
```

```
## Type III Analysis of Variance Table with Satterthwaite's method
##           Sum Sq Mean Sq NumDF DenDF F value Pr(>F)    
## Genre         39     9.9     4   271    2.66  0.033 *  
## PlayType2      0     0.0     1   186    0.00  0.944    
## f_period3    474   236.8     2   230   63.85 <2e-16 ***
## ---
## Signif. codes:  0 '***' 0.001 '**' 0.01 '*' 0.05 '.' 0.1 ' ' 1
```

```
# Fit the normal model with weights based on the normal distribution model absolute residuals
plays4$normal_weights3f = 1/((-0.2055 + 0.2458*predict(mod1a_final))^2)

# Final model - using weights to adjust for non-constant variability in the residuals
mod1_final<-lmer(mode~ Genre + PlayType2 + f_period3 + (1|Author2),weight = normal_weights3f,data=plays4,REML=F)
summary(mod1_final)
```

```
## Linear mixed model fit by maximum likelihood . t-tests use Satterthwaite's method ['lmerModLmerTest']
## Formula: mode ~ Genre + PlayType2 + f_period3 + (1 | Author2)
##    Data: plays4
## Weights: normal_weights3f
## 
##      AIC      BIC   logLik deviance df.resid 
##   1094.1   1130.1   -537.0   1074.1      263 
## 
## Scaled residuals: 
##    Min     1Q Median     3Q    Max 
## -2.791 -0.524 -0.119  0.349  3.601 
## 
## Random effects:
##  Groups   Name        Variance Std.Dev.
##  Author2  (Intercept) 0.127    0.356   
##  Residual             1.935    1.391   
## Number of obs: 273, groups:  Author2, 31
## 
## Fixed effects:
##                    Estimate Std. Error       df t value Pr(>|t|)    
## (Intercept)          8.4006     0.3752 229.3674   22.39  < 2e-16 ***
## GenreHistory         1.1555     0.4437 272.7185    2.60   0.0097 ** 
## GenreMisc            0.1626     0.3859 266.9199    0.42   0.6738    
## GenreTragedy        -0.4997     0.2334 265.6766   -2.14   0.0331 *  
## GenreTragicomedy    -0.6122     0.3055 259.9336   -2.00   0.0461 *  
## PlayType2Other       0.0807     0.2401 141.0485    0.34   0.7372    
## f_period31597-1602  -2.5641     0.4283 271.0074   -5.99  6.8e-09 ***
## f_period3> 1602     -3.7018     0.3562 250.6978  -10.39  < 2e-16 ***
## ---
## Signif. codes:  0 '***' 0.001 '**' 0.01 '*' 0.05 '.' 0.1 ' ' 1
## 
## Correlation of Fixed Effects:
##             (Intr) GnrHst GnrMsc GnrTrgd GnrTrgc PlyT2O f_3159
## GenreHistry -0.286                                            
## GenreMisc   -0.385  0.149                                     
## GenreTragdy -0.264  0.228  0.238                              
## GenrTrgcmdy -0.184  0.179  0.163  0.359                       
## PlyTyp2Othr -0.257  0.187 -0.055  0.149   0.163               
## f_31597-160 -0.681 -0.034  0.266  0.046   0.013  -0.021       
## f_prd3>1602 -0.853  0.142  0.266 -0.039  -0.068   0.036  0.706
```

```
anova(mod1_final)
```

```
## Type III Analysis of Variance Table with Satterthwaite's method
##           Sum Sq Mean Sq NumDF DenDF F value Pr(>F)    
## Genre       35.0     8.7     4   269    4.51 0.0015 ** 
## PlayType2    0.2     0.2     1   141    0.11 0.7372    
## f_period3  216.0   108.0     2   260   55.81 <2e-16 ***
## ---
## Signif. codes:  0 '***' 0.001 '**' 0.01 '*' 0.05 '.' 0.1 ' ' 1
```

```
# Poisson model with identity link for comparison
mod1_final_p<-glmer(mode~ Genre + PlayType2 + f_period3 + (1|Author2),family=poisson(link = "identity"),data=plays4)
```

```
## boundary (singular) fit: see ?isSingular
```

```
summary(mod1_final_p)
```

```
## Generalized linear mixed model fit by maximum likelihood (Laplace Approximation) ['glmerMod']
##  Family: poisson  ( identity )
## Formula: mode ~ Genre + PlayType2 + f_period3 + (1 | Author2)
##    Data: plays4
## 
##      AIC      BIC   logLik deviance df.resid 
##   1152.4   1184.8   -567.2   1134.4      264 
## 
## Scaled residuals: 
##    Min     1Q Median     3Q    Max 
## -2.657 -0.361 -0.161  0.296  3.027 
## 
## Random effects:
##  Groups  Name        Variance Std.Dev.
##  Author2 (Intercept) 1e-14    1e-07   
## Number of obs: 273, groups:  Author2, 31
## 
## Fixed effects:
##                    Estimate Std. Error z value Pr(>|z|)    
## (Intercept)          8.5614     0.4432   19.32  < 2e-16 ***
## GenreHistory         1.0953     0.5650    1.94    0.053 .  
## GenreMisc            0.3843     0.5187    0.74    0.459    
## GenreTragedy        -0.4066     0.3599   -1.13    0.259    
## GenreTragicomedy    -0.6313     0.4994   -1.26    0.206    
## PlayType2Other       0.0487     0.3306    0.15    0.883    
## f_period31597-1602  -2.6658     0.5221   -5.11  3.3e-07 ***
## f_period3> 1602     -3.8192     0.4127   -9.25  < 2e-16 ***
## ---
## Signif. codes:  0 '***' 0.001 '**' 0.01 '*' 0.05 '.' 0.1 ' ' 1
## 
## Correlation of Fixed Effects:
##             (Intr) GnrHst GnrMsc GnrTrgd GnrTrgc PlyT2O f_3159
## GenreHistry -0.392                                            
## GenreMisc   -0.525  0.220                                     
## GenreTragdy -0.380  0.252  0.286                              
## GenrTrgcmdy -0.232  0.181  0.180  0.297                       
## PlyTyp2Othr -0.314  0.248  0.029  0.118   0.151               
## f_31597-160 -0.612 -0.032  0.314  0.104   0.027  -0.026       
## f_prd3>1602 -0.803  0.205  0.359  0.029  -0.057   0.048  0.607
## optimizer (Nelder_Mead) convergence code: 0 (OK)
## boundary (singular) fit: see ?isSingular
```

```
# Model goodness of fit summary
cbind(model = c("Normal no SD function","Poisson","Normal with SD function"),AIC = AIC(mod1a_final,mod1_final_p,mod1_final))
```

```
##                                model AIC.df AIC.AIC
## mod1a_final    Normal no SD function     10  1167.7
## mod1_final_p                 Poisson      9  1152.4
## mod1_final   Normal with SD function     10  1094.1
```

### Model assumptions - residuals and random effects - final model

S2 Fig 17 Model diagnostics for the final model of the mode using the
Normal distribution model with weights based on an SD function, 2 plays
removed from the data

S2 Fig 18 Random effects distribution for the final model of the mode
using the Normal distribution model with weights based on an SD
function, 2 plays removed from the data

# Conclusion

From this analysis it was determined that a normal distribution model
with an appropriate weighting function was a substantially better fit
(AIC 1094) to the mode data, AIC 58 lower than a Poisson model (AIC
1152). Therefore the normal model will be used for the mode analysis
with an SD function based on the iterative scheme without the play
Philotas.

The plays Tragedy of Miriam, an extreme outlier with mode 33 will be
dropped from the data set prior to analysis and the play Philotas an
extreme outlier with Pearson residual of 9, will be removed from all
subsequent analyses as not being typical of the plays produced in that
era.

# References

Burnham, K.P. and Anderson, D. R., 2004, Multimodel Inference
Understanding AIC and BIC in Model Selection, Sociological Methods &
Research, Vol. 33, No. 2, 261-304, Sage Publications, 2004.

Davidian, M. and Carroll, R. J., 1987, Variance Function Estimation,
Journal of the American Statistical Association, 82,1079-1091.

# Appendix - Pearson residuals in diagnostic plots

The appendix records the investigation to understand how to specify
model residuals using the residuals(.merMod) function.

## The Normal distribution case

The usual choices with direct calls from the R residuals function for
use in diagnostic plots needed to be examined so that correct choices
could be setup in the model\_diagnostics() function used in this
analysis. There are 2 important options in the residual function, type
of residual, the focus here will be on response and Pearson, and whether
scaling is used or not. The complication being the introduction of the
weighting scheme based on an SD function.

The summary of what follows is that when model weights are used based
on an SD function the scaling option in the residuals function should be
set to unscaled (scaled = F). When there is no weighting then the
Pearson residual scaling option should be set (scaled = T). Choosing
Pearson residuals without scaling leads to the same as response
residuals.

```
# Check on Pearson residual calculations methods used in the model_diagnostics function
# Actual vs not scaled and how do these work with or without a weighting function

# From the lme4 help on the residuals.merMod function
# The lmer module is used for the normal distribution model and glmer for the Poisson model.
# From the help file we read the following.
# 
# The default residual type varies between lmerMod and glmerMod objects: they
# try to mimic residuals.lm and residuals.glm respectively. In particular, the
# default type is "response", i.e. (observed-fitted) for lmerMod objects vs.
# "deviance" for glmerMod objects. type="partial" is not yet implemented for
# either type.
# 
#   ## S3 method for class 'merMod'  
#  residuals(object,  
#     type = if (isGLMM(object)) "deviance" else "response",  
#     scaled = FALSE, ...)  
# 
#   ## S3 method for class 'lmResp'  
#  residuals(object,  
#     type = c("working", "response", "deviance", "pearson", "partial"),  
#     ...)  
#   ## S3 method for class 'glmResp'  
#  residuals(object,  
#     type = c("deviance", "pearson", "working", "response", "partial"),  
#     ...)  

# Model without weights
mod0<-lmer(mode~ Genre + PlayType2 + f_period3 + (1|Author2),data=plays3,REML=F)
#summary(mod0)

# Add weights to the next model and refit
plays3$w = 1/((-0.097 + 0.2384*predict(mod0))^2)

# model using weights to adjust for non-constant variability in the residuals
mod1<-lmer(mode~ Genre + PlayType2 + f_period3 + (1|Author2),weight = w,data=plays3,REML=F)
#summary(mod1)

# How do the different Pearson residual options operate with and without weighting?
# Firstly a model with weighting
mod1_resid = data.frame(resid_response_no_scale=residuals(mod1,scaled=F,type="response"),
                        resid_pearson_no_scale=residuals(mod1,scaled=F,type="pearson"),
                        resid_pearson_scaled=residuals(mod1,scaled=T,type="pearson"),
                        pred=predict(mod1),w=plays3$w)
# Add additional residual calculations
mod1_resid = mod1_resid %>% mutate(residual_sigma = sigma(mod1),
                                   resid_pearson_no_scale_sigma = resid_pearson_no_scale/residual_sigma,
                                   resid_weighted = resid_response_no_scale/(-0.097 +0.2384*predict(mod0)),
                                   resid_weighted2 = resid_response_no_scale*sqrt(w),
                                   )
head(mod1_resid)
```

```
##   resid_response_no_scale resid_pearson_no_scale resid_pearson_scaled   pred       w residual_sigma
## 1               -0.053744              -0.059678            -0.042565 4.0537 1.23302         1.4021
## 2                2.140199               1.423168             1.015055 6.8598 0.44219         1.4021
## 3               -0.542751              -0.545958            -0.389397 4.5428 1.01185         1.4021
## 4               -0.627854              -0.430199            -0.306833 6.6279 0.46948         1.4021
## 5               -1.399323              -0.674360            -0.480977 9.3993 0.23225         1.4021
## 6                3.246460               2.635060             1.879419 5.7535 0.65881         1.4021
##   resid_pearson_no_scale_sigma resid_weighted resid_weighted2
## 1                    -0.042565      -0.059678       -0.059678
## 2                     1.015055       1.423168        1.423168
## 3                    -0.389397      -0.545958       -0.545958
## 4                    -0.306833      -0.430199       -0.430199
## 5                    -0.480977      -0.674360       -0.674360
## 6                     1.879419       2.635060        2.635060
```

```
# Correct for the weighted model
S2_Fig_20 = model_diagnostics(plays3,mod1,"Model weights used",ModelWeightsUsed = T)
```

S2 Fig 19 Model diagnostics for the weighted model

S2 Fig 21 Model diagnostics for the unweighted model

### Firstly a model with weights

For Pearson residuals when weighting is used the calculation is based
on (response residual)/(SD from weighting function) without applying
scaling from the R residual function (residuals.merMod). In the data
frame above various options have been calculated and are examined
here.

Firstly resid\_response\_no\_scale is the raw residual based on
(data-predicted) with no scaling adjustment using the code
residuals(mod1,scaled=F,type=“response”).

A Pearson residual without scaling resid\_pearson\_no\_scale is the
Pearson residual without scaling, code
residuals(mod1,scaled=F,type=“pearson”). They differ due to the SD
function having been used through the model weights. This is confirmed
by calculation of additional columns resid\_weighted and resid\_weighted2
that agree with the values in this column by dividing the response
residual by the SD function of the weight^1/2$ that is equivalent to the
SD function. Therefore when weighting is being used the correct residual
call is without scaling  
residuals(mod1,scaled=F,type=“pearson”)

An alternative option that is not correct is to request the Pearson
residual with scaling, resid\_pearson\_scaled. This does not agree with
the calculation above. Instead it leads to smaller residuals than
resid\_pearson\_no\_scale due to dividing by the model residual SD, in this
case the value being 1.4413 (see column residual\_sigma and mod1 summary
output). This is confirmed in the variable resid\_pearson\_no\_scale\_sigma
in which the unscaled Pearson residual is divided by the residual SD and
gives the same values as the residual function call  
residuals(mod1,scaled=T,type=“pearson”)

The conclusion being that when weights are used based on an SD
function the correct Pearson residual call is without scaling,
residuals(mod1,scaled=F,type=“response”).

### when the model does not use weights

```
mod0<-lmer(mode~ Genre + PlayType2 + f_period3 + (1|Author2),data=plays3,REML=F)
summary(mod0)
```

```
## Linear mixed model fit by maximum likelihood . t-tests use Satterthwaite's method ['lmerModLmerTest']
## Formula: mode ~ Genre + PlayType2 + f_period3 + (1 | Author2)
##    Data: plays3
## 
##      AIC      BIC   logLik deviance df.resid 
##   1202.6   1238.7   -591.3   1182.6      264 
## 
## Scaled residuals: 
##    Min     1Q Median     3Q    Max 
## -3.716 -0.474 -0.196  0.279  5.151 
## 
## Random effects:
##  Groups   Name        Variance Std.Dev.
##  Author2  (Intercept) 0.435    0.66    
##  Residual             4.114    2.03    
## Number of obs: 274, groups:  Author2, 32
## 
## Fixed effects:
##                    Estimate Std. Error      df t value Pr(>|t|)    
## (Intercept)           8.360      0.379 113.165   22.03  < 2e-16 ***
## GenreHistory          0.998      0.456 271.007    2.19    0.029 *  
## GenreMisc             0.350      0.417 272.954    0.84    0.402    
## GenreTragedy         -0.252      0.334 273.408   -0.75    0.451    
## GenreTragicomedy     -0.505      0.491 273.912   -1.03    0.304    
## PlayType2Other        0.177      0.317 179.022    0.56    0.577    
## f_period31597-1602   -2.469      0.422 257.324   -5.85  1.5e-08 ***
## f_period3> 1602      -3.610      0.351 169.508  -10.29  < 2e-16 ***
## ---
## Signif. codes:  0 '***' 0.001 '**' 0.01 '*' 0.05 '.' 0.1 ' ' 1
## 
## Correlation of Fixed Effects:
##             (Intr) GnrHst GnrMsc GnrTrgd GnrTrgc PlyT2O f_3159
## GenreHistry -0.400                                            
## GenreMisc   -0.481  0.289                                     
## GenreTragdy -0.340  0.287  0.303                              
## GenrTrgcmdy -0.209  0.195  0.186  0.288                       
## PlyTyp2Othr -0.348  0.218  0.024  0.107   0.109               
## f_31597-160 -0.488 -0.054  0.260  0.056   0.010   0.000       
## f_prd3>1602 -0.671  0.151  0.260 -0.045  -0.087   0.061  0.516
```

```
# How do the different Pearson residual options operate without weighting?

mod0_resid = data.frame(resid_response_no_scale=residuals(mod0,scaled=F,type="response"),
                        resid_pearson_no_scale=residuals(mod0,scaled=F,type="pearson"),
                        resid_pearson_scaled=residuals(mod0,scaled=T,type="pearson"),
                        pred=predict(mod0))
# Add additional residual calculations
mod0_resid = mod0_resid %>% mutate(residual_sigma = sigma(mod0),
                                   resid_pearson_no_scale_sigma = resid_response_no_scale/residual_sigma)
head(mod0_resid)
```

```
##   resid_response_no_scale resid_pearson_no_scale resid_pearson_scaled   pred residual_sigma
## 1                -0.18441               -0.18441            -0.090924 4.1844         2.0282
## 2                 2.28512                2.28512             1.126677 6.7149         2.0282
## 3                -0.57687               -0.57687            -0.284424 4.5769         2.0282
## 4                -0.52874               -0.52874            -0.260695 6.5287         2.0282
## 5                -1.11090               -1.11090            -0.547730 9.1109         2.0282
## 6                 3.42523                3.42523             1.688805 5.5748         2.0282
##   resid_pearson_no_scale_sigma
## 1                    -0.090924
## 2                     1.126677
## 3                    -0.284424
## 4                    -0.260695
## 5                    -0.547730
## 6                     1.688805
```

In the case of no weights being used the Pearson residual without
scaling is the same as the response residual. Only when scaling is used
is the response residual divided by the residual SD, in the case of mod0
being 2.0846.

### Conclusion about residuals in the normal case

For Pearson residuals the scaling option needs to be set in
accordance with whether weights are used in the model or not.

When weights are used in the model the call should be without
scaling, e.g.  
residuals(mod1,scaled=F,type=“pearson”)

When weights are not used in the model the call should use scaling,
e.g.  
residuals(mod0,scaled=T,type=“pearson”)

## The Poisson distribution case

When the Poisson distribution is used there is an inbuilt variance
function and setting Pearson residuals as if there was no weighting,
i.e. no scaling should be correct. Actually setting scaling = T gives
the same answer. As it turns out it doesn’t matter what option is chosen
for the Poisson model, scaled or unscaled, the Pearson residuals are the
same. Also no difference for identify or log link functions.

```
# Model without weights
mod1p<-glmer(mode~ Genre + PlayType2 + f_period3 + (1|Author2),family=poisson(link="identity"),data=plays)
summary(mod1p)
```

```
## Generalized linear mixed model fit by maximum likelihood (Laplace Approximation) ['glmerMod']
##  Family: poisson  ( identity )
## Formula: mode ~ Genre + PlayType2 + f_period3 + (1 | Author2)
##    Data: plays
## 
##      AIC      BIC   logLik deviance df.resid 
##   1245.4   1277.9   -613.7   1227.4      266 
## 
## Scaled residuals: 
##    Min     1Q Median     3Q    Max 
## -2.557 -0.490 -0.214  0.255 11.324 
## 
## Random effects:
##  Groups  Name        Variance Std.Dev.
##  Author2 (Intercept) 0.156    0.395   
## Number of obs: 275, groups:  Author2, 32
## 
## Fixed effects:
##                    Estimate Std. Error z value Pr(>|z|)    
## (Intercept)           8.098      0.458   17.67  < 2e-16 ***
## GenreHistory          1.346      0.579    2.33    0.020 *  
## GenreMisc             0.394      0.538    0.73    0.463    
## GenreTragedy          0.144      0.389    0.37    0.711    
## GenreTragicomedy     -0.535      0.510   -1.05    0.294    
## PlayType2Other        0.622      0.354    1.76    0.079 .  
## f_period31597-1602   -2.525      0.542   -4.66  3.2e-06 ***
## f_period3> 1602      -3.483      0.441   -7.90  2.8e-15 ***
## ---
## Signif. codes:  0 '***' 0.001 '**' 0.01 '*' 0.05 '.' 0.1 ' ' 1
## 
## Correlation of Fixed Effects:
##             (Intr) GnrHst GnrMsc GnrTrgd GnrTrgc PlyT2O f_3159
## GenreHistry -0.341                                            
## GenreMisc   -0.489  0.240                                     
## GenreTragdy -0.272  0.242  0.293                              
## GenrTrgcmdy -0.209  0.174  0.170  0.283                       
## PlyTyp2Othr -0.261  0.207  0.028  0.055   0.133               
## f_31597-160 -0.613 -0.058  0.276  0.039   0.026  -0.043       
## f_prd3>1602 -0.787  0.149  0.307 -0.064  -0.078   0.010  0.612
```

```
# How do the different Pearson residual options operate with and without weighting?
# Firstly a model with weighting
mod1p_resid = data.frame(resid_response_no_scale=residuals(mod1p,scaled=F,type="response"),
                        resid_pearson_no_scale=residuals(mod1p,scaled=F,type="pearson"),
                        resid_pearson_scaled=residuals(mod1p,scaled=T,type="pearson"),
                        pred=predict(mod1p))

# Add additional residual calculations
mod1p_resid = mod1p_resid %>% mutate(w=1/pred,
                                   residual_sigma = sigma(mod1p),
                                   resid_pearson_no_scale_sigma = resid_pearson_no_scale/residual_sigma,
                                   resid_weighted = resid_response_no_scale/sqrt(pred),
                                   resid_weighted2 = resid_response_no_scale*sqrt(w))
head(mod1p_resid)
```

```
##   resid_response_no_scale resid_pearson_no_scale resid_pearson_scaled   pred       w residual_sigma
## 1                -0.60351               -0.28128             -0.28128 4.6035 0.21723              1
## 2                 2.15243                0.82255              0.82255 6.8476 0.14604              1
## 3                -0.54410               -0.25524             -0.25524 4.5441 0.22007              1
## 4                -0.70665               -0.27287             -0.27287 6.7066 0.14911              1
## 5                -1.39113               -0.45395             -0.45395 9.3911 0.10648              1
## 6                 3.11008                1.28149              1.28149 5.8899 0.16978              1
##   resid_pearson_no_scale_sigma resid_weighted resid_weighted2
## 1                     -0.28128       -0.28128        -0.28128
## 2                      0.82255        0.82255         0.82255
## 3                     -0.25524       -0.25524        -0.25524
## 4                     -0.27287       -0.27287        -0.27287
## 5                     -0.45395       -0.45395        -0.45395
## 6                      1.28149        1.28149         1.28149
```

```
# What about log link?
mod1pl<-glmer(mode~ Genre + PlayType2 + f_period3 + (1|Author2),family=poisson(link="log"),data=plays)
summary(mod1pl)
```

```
## Generalized linear mixed model fit by maximum likelihood (Laplace Approximation) ['glmerMod']
##  Family: poisson  ( log )
## Formula: mode ~ Genre + PlayType2 + f_period3 + (1 | Author2)
##    Data: plays
## 
##      AIC      BIC   logLik deviance df.resid 
##   1247.1   1279.6   -614.5   1229.1      266 
## 
## Scaled residuals: 
##    Min     1Q Median     3Q    Max 
## -2.524 -0.476 -0.241  0.235 11.435 
## 
## Random effects:
##  Groups  Name        Variance Std.Dev.
##  Author2 (Intercept) 0.0064   0.08    
## Number of obs: 275, groups:  Author2, 32
## 
## Fixed effects:
##                    Estimate Std. Error z value Pr(>|z|)    
## (Intercept)          2.0530     0.0678   30.27  < 2e-16 ***
## GenreHistory         0.1825     0.0828    2.20    0.028 *  
## GenreMisc            0.0721     0.0766    0.94    0.347    
## GenreTragedy         0.0285     0.0674    0.42    0.672    
## GenreTragicomedy    -0.1186     0.1079   -1.10    0.271    
## PlayType2Other       0.0866     0.0592    1.46    0.143    
## f_period31597-1602  -0.3254     0.0778   -4.18  2.9e-05 ***
## f_period3> 1602     -0.5041     0.0642   -7.85  4.3e-15 ***
## ---
## Signif. codes:  0 '***' 0.001 '**' 0.01 '*' 0.05 '.' 0.1 ' ' 1
## 
## Correlation of Fixed Effects:
##             (Intr) GnrHst GnrMsc GnrTrgd GnrTrgc PlyT2O f_3159
## GenreHistry -0.471                                            
## GenreMisc   -0.532  0.357                                     
## GenreTragdy -0.360  0.321  0.358                              
## GenrTrgcmdy -0.217  0.189  0.189  0.254                       
## PlyTyp2Othr -0.397  0.250  0.034  0.058   0.087               
## f_31597-160 -0.457 -0.042  0.251  0.052   0.013   0.015       
## f_prd3>1602 -0.638  0.179  0.267 -0.054  -0.086   0.092  0.441
```

```
# With the log link residuals and predicted are on the log scale
mod1pl_resid = data.frame(resid_response_no_scale=residuals(mod1pl,scaled=F,type="response"),
                        resid_pearson_no_scale=residuals(mod1pl,scaled=F,type="pearson"),
                        resid_pearson_scaled=residuals(mod1pl,scaled=T,type="pearson"),
                        pred=predict(mod1pl))

# Add additional residual calculations - not interested in comparing response residuals as have to 
# back transform from the log scale 
mod1pl_resid = mod1pl_resid %>% mutate(w=1/pred,
                                   residual_sigma = sigma(mod1pl),
                                   resid_pearson_no_scale_sigma = resid_pearson_no_scale/residual_sigma)
head(mod1pl_resid)
```

```
##   resid_response_no_scale resid_pearson_no_scale resid_pearson_scaled   pred       w residual_sigma
## 1                -0.68726               -0.31744             -0.31744 1.5448 0.64731              1
## 2                 2.34756                0.91018              0.91018 1.8950 0.52771              1
## 3                -0.63586               -0.29532             -0.29532 1.5338 0.65197              1
## 4                -0.55233               -0.21577             -0.21577 1.8798 0.53197              1
## 5                -1.19746               -0.39484             -0.39484 2.2189 0.45067              1
## 6                 3.43612                1.45673              1.45673 1.7163 0.58265              1
##   resid_pearson_no_scale_sigma
## 1                     -0.31744
## 2                      0.91018
## 3                     -0.29532
## 4                     -0.21577
## 5                     -0.39484
## 6                      1.45673
```
