# Supplementary material for: Changes in the length of speeches in the plays of William Shakespeare and his contemporaries: A mixed models approach
Source: PLoS One. 2023 Apr 21;18(4):e0282716. doi: 10.1371/journal.pone.0282716 (PMC10121026; doi:10.1371/journal.pone.0282716)
Supplement: S3 File — (HTML) [file pone.0282716.s003.html]

S3-Speech-length-mode-adjustment.knit


### Changes in the length of speeches in the plays of William Shakespeare and his contemporaries: a mixed models approach

Colyvas, Craig and Egan

# Supplementary 3 Speech length mode adjustment

The possibility of multiple peaks within a play having the same
frequency was examined as this would mean a confilct as to which one
should be considered the mode.

# Look for modes that are not unique

```
library(tidyverse)
```

```
## Warning: package 'tidyverse' was built under R version 4.1.3
```

```
## -- Attaching packages --------------------------------------- tidyverse 1.3.1 --
```

```
## v ggplot2 3.3.6     v purrr   0.3.4
## v tibble  3.1.6     v dplyr   1.0.8
## v tidyr   1.1.4     v stringr 1.4.0
## v readr   2.1.1     v forcats 0.5.1
```

```
## Warning: package 'ggplot2' was built under R version 4.1.3
```

```
## -- Conflicts ------------------------------------------ tidyverse_conflicts() --
## x dplyr::filter() masks stats::filter()
## x dplyr::lag()    masks stats::lag()
```

```
###Read the primary data
data1=read.table("Speeches.txt",header=T,sep="\t",row.names = NULL,quote = "" )

# Mode for each play - first arrange the frequency for each speech length from the most frequent to least and save the most frequent as the mode
mode1=data1 %>%group_by(TextTitle,Speech_Word_Count)%>%summarise(freq=n())%>%arrange(TextTitle,desc(freq))%>%summarise(mode=first(Speech_Word_Count),freq=first(freq))
```

```
## `summarise()` has grouped output by 'TextTitle'. You can override using the
## `.groups` argument.
```

```
# This time save the second most frequent speech length
mode2=data1 %>%group_by(TextTitle,Speech_Word_Count)%>%summarise(freq=n())%>%arrange(TextTitle,desc(freq))%>%summarise(mode2=nth(Speech_Word_Count,2),freq2=nth(freq,2))
```

```
## `summarise()` has grouped output by 'TextTitle'. You can override using the
## `.groups` argument.
```

```
# and the third most frequent
mode3=data1 %>%group_by(TextTitle,Speech_Word_Count)%>%summarise(freq=n())%>%arrange(TextTitle,desc(freq))%>%summarise(mode3=nth(Speech_Word_Count,3),freq3=nth(freq,3))
```

```
## `summarise()` has grouped output by 'TextTitle'. You can override using the
## `.groups` argument.
```

```
# and the 4th most frequent
mode4=data1 %>%group_by(TextTitle,Speech_Word_Count)%>%summarise(freq=n())%>%arrange(TextTitle,desc(freq))%>%summarise(mode4=nth(Speech_Word_Count,4),freq4=nth(freq,4))
```

```
## `summarise()` has grouped output by 'TextTitle'. You can override using the
## `.groups` argument.
```

```
# Combine the 4 versions of possible modes
check_mode = cbind(mode1,mode2[,-1],mode3[,-1],mode4[,-1])

# Add the mode information about multiple modes to the speeches data set
speeches = left_join(data1,check_mode,by="TextTitle")

# Keep only the plays with frequency of the first peak equal to the freq of the second peak
play1 = filter(speeches, freq == freq2)
```

S3 Fig 1 Distribution of lengths of speeches (range 1 to 30). The red
vertical marker indicates the option opton chosen by the automatic
procedure. The blue marker the next largest speech length that was just
as common.

The histograms in S3 Fig 1 show the distributions of the plays that
had 2 or more peaks with the same height. The peaks with equal height
are shown with red (the first chosen mode) and in blue (the second peak
with equal height but greater speech length).

```
options(width=110)
# Check to see how many of the secondary peaks have the  same frequency the first

# No plays with 4 speech lengths equally likely
filter(check_mode,freq==freq2 & freq == freq3 & freq == freq4)
```

```
## [1] TextTitle mode      freq      mode2     freq2     mode3     freq3     mode4     freq4    
## <0 rows> (or 0-length row.names)
```

```
# 1 play with 3 speech lengths equally likely
filter(check_mode,freq==freq2 & freq == freq3 )
```

```
##         TextTitle mode freq mode2 freq2 mode3 freq3 mode4 freq4
## 1 Edmond Ironside    4   15     8    15     9    15    10    14
```

```
# 15 plays with 2 speech lengths equally likely
filter(check_mode,freq==freq2 )
```

```
##                           TextTitle mode freq mode2 freq2 mode3 freq3 mode4 freq4
## 1                1 Henry the Fourth    6   42     8    42     9    41     4    38
## 2         All's Well That Ends Well    4   61     5    61    10    52     8    45
## 3          Alphonsus King of Aragon    8    9    48     9     9     8    34     7
## 4                    Bussy D'Ambois    4   33     5    33     3    27     9    26
## 5                         Cleopatra    7    9    10     9     9     7     6     6
## 6                   Edmond Ironside    4   15     8    15     9    15    10    14
## 7              Faithful Shepherdess    5   12    16    12     1    11     2    11
## 8                         Gallathea    6   25     8    25     3    24     7    24
## 9                 Massacre at Paris    4   22     8    22     7    20     9    20
## 10                          May Day    5   59     6    59     4    58     8    51
## 11           Much Ado About Nothing    6   66     9    66     7    61     8    57
## 12              Tancred and Gismund    7   16     8    16     4    10     6    10
## 13 Tragedy of Charles Duke of Byron    4   21     9    21     3    17     5    14
## 14                    What You Will    4   44     9    44     3    42     6    38
## 15                    Widow's Tears    4   54     5    54     3    46     6    43
```

In the table above there 15 plays out of the 275 that had multiple
peaks with equal height, 14 with two equal peaks and 1 with 3 equal
peaks.

The procedure used to determine the mode for each play was to count
the number of speeches of each length and then to sort the speeches in
descending order and choose the most commonly occurring speech length as
the mode. This is the column headed mode in the data table above and its
associated frequency is in column freq. However this procedure could not
separate between two (or more) speech lengths with the same frequency,
these being identified in the column mode2 and freq2 and mode3 and freq3
for Edmund Ironside. The shortest speech length was chosen by this
procedure so this could create a bias to lower modes for speech length.
A way to resolve this was sought.

Option 1: Carry out a sensitivity analysis with the lower mode and
repeat the analysis with the higher mode and see if the results would be
changed. This was not appealing, the option below was chosen
instead.

Option 2: Use the distribution of the data as a guide to assist in
choosing a most probable mode. The reasoning behind this is the mode is
usually associated with the area of the distribution with the highest
density. So peaks close to the highest density would be favoured over
those that were distant from this part of the distribution. These
distant ones being considered more likely just an artefact of natural
variability in speech lengths.

### Mode not changed

This covers the logic behind why the mode for these plays was kept at
the original choice.

1. For the play Alphonsus King of Aragon where the second mode
   possibility was 48 choosing the lower mode was the obvious choice as
   there was much less probability density at the higher speech
   lengths.
2. Not as extreme but in a similar vein there were two plays where
   the lower peak was associated with more density and seemed a more likely
   option. These were Much Ado About Nothing and the Tragedy of Charles
   Duke of Byron.
3. Modes that were close together but one was probably a little
   closer the higher density part of the distribution: All’s Well That Ends
   Well, Bussy D’Ambois, Faithful Shepherdess, Gallathea, May Day, Tancred
   and Gismund and Widow’s Tears.
4. A little less clear were plays in which the modes were more
   widely separated but still appeared close to the highest density region.
   In these case however the lower mode was again favoured, 1 Henry the
   Fourth, Cleopatra and What You Will

### Mode changed

5. However, for a play like Edmond Ironside the lower mode appeared
   less likely as it was more like a single isolated peak and the greater
   density was at the higher value 8. In this case changing the mode to the
   higher value of 8 would seem to be more appropriate. Similarly for
   Massacre at Paris.

# Conclusion

After evaluating the distributions for the 15 plays with 2 or more
peaks with equal height only 2 plays were considered needing a change in
the mode.

Edmond Ironside: change mode from 4 to 8  
Massacre at Paris: change mode from 4 to 8

The plot below shows the final assignments for the modes for these 15
plays.

```
# Modify modes for these 2 plays
play1$mode[play1$TextTitle == "Edmond Ironside"] = 8
play1$mode[play1$TextTitle == "Massacre at Paris"] = 8
```

S3 Fig 2 Distribution of lengths of speeches (range 1 to 30). The red
vertical marker indicates the final choice for mode.
