# Supplementary material for: Changes in the length of speeches in the plays of William Shakespeare and his contemporaries: A mixed models approach
Source: PLoS One. 2023 Apr 21;18(4):e0282716. doi: 10.1371/journal.pone.0282716 (PMC10121026; doi:10.1371/journal.pone.0282716)
Supplement: Attachment — Submitted filename: Response to reviewers-KC Hugh.docx [file pone.0282716.s009.docx]

Major concerns:

1. The innovative centrepiece of the paper is the application of the linear mixed model (LME) to demonstrate that the reduction of the average number of words per sentence in the play is robust with respect to other contributing factors, such as authorship, genre, theater, etc. To my surprise, I did not find any report of the LME model these claims are based on. This model along with all parameters and its statistical characteristics must be displayed in the main body of the paper accompanied with detailed explanation. The authors must explain all variables and justify the choice of the random effect. Note that the discussion of the model in SI part is not sufficient. Moreover, it seems that the authors present many models in SI, but it’s unclear what model is the final for interpreting the results and drawing conclusions in the abstract. Generally, I would like to have more detailed description of the methodology – the driving force of the results of the work.

**Response**
Requested to provide more detail about the methodology and the LME model.

- Mixed model parameter and statistical characteristics with detailed explanation
  A section has been added following the statistical methods section describing aspects of the mixed model. Keeping in mind the likely readership of the paper the mathematical components were kept to a minimum, trying to focus more on the concepts.
- Explain all variables

Section 2 has been reorganised and amplified to describe each variable in turn.

- Justify author as a random effect
  Justification was added to the new mixed model section and supported by showing in section 3.4 Speech length by author how the author random effect could be applied to make inference about the wider population of authors.
- Many models have been fitted and
  what model is the final for interpreting the results and drawing conclusions in the abstract
  The problem of many models in S1 has been reduced considerably. Most of the alternative models were moved to S2 as part of the investigation comparing the normal model and the Poisson. Resulting from that one final model in S1 was used as the foundation for reporting results in the manuscript. Small variations on that model in S1 were minor (test interactions, repeat with data reduced to one play type) and based on the final model.

2. The nonlinear curves derived through nonparametric estimation via splines displayed in Fig. 1, 5, etc. are not justified. They look to me as the result of overfitting – a typical artifact unless they can be explained and tested through extensive simulations. Instead, just using a simpler but more robust two-segment regression would be more trustful.

**Response**
These kind of smoothing curves are to assist the viewer in detecting patterns in the data. The one chosen (lowess) is widely used for this purpose and has reasonable properties not being too reactive to the data. A less responsive smoothing method has been chosen to replace it based on Generalised Additive Models (GAM). Again, the only purpose was to guide the reader as to any general pattens and pick up any curvature that might be present in the data as opposed to forcing a linear relationship interpretation. Any statements about what might be significant would be supported by formal statistical tests after clues from the plots alerted the investigator to possible patterns in the data.

3. The authors should explain or at least offer some hypothesis why sentences became dramatically shorter within a 5-year period. What happened? That would considerably increase the visibility of the work.

**Response**
We have amplified the section on the literary and theatrical context for the observed changes (4.2) to provide hypotheses for causation, i.e. the authors’ wish to make onstage dialogue more like natural speech; a collective adoption of a new fashion; and an authorial wish for more lively dramatic dialogue.

4. It is established in statistics to use Poisson distribution for counts such as the number of words in the sentence. I suggest reconsidering the model using log link in the GLM mixed model. Alternatively, the authors may work with log-transformed counts. This approach will substantially reduce the effect of outliers.

**Response**
The Poisson distribution would have been most directly applicable to the original speech length data where the measure was a count. However, the data forming most of the analyses was based on summary statistics from each play, mode, mean and median. Summary statistics would be expected to have different properties to the original measure due to condensing the information in the whole distribution into a single value.

The suggestion to use the Poisson distribution was not unreasonable however due to the discrete nature of the data so Poisson models were evaluated against the normal model. Initially it appeared the Poisson was the better choice. In seeking to understand why it was better some important issues were discovered to do with influential observations and non-constant variance in the residuals. The investigation developed into a very substantial side investigation that is documented in an additional supplementary file (S2). Eventually the normal distribution was found to be a better choice than the Poisson, but the quality of the normal model was improved over the original version because of what was found. A summary describing more details about this and what was done provided is at the beginning of S2.

5. I see lack of statistical significance testing and reporting p-values when comparing the results from two time periods. These p-values should be displayed right on the figures.

**Response**
Letter codes have been added to the plots with CIs to indicate which categories are statistically significantly different. The use of these in interpretation is supported by text in the figure captions and the manuscript text.

Minor concerns:

1. Replace “English plays (1538-1642)” with “…plays of William Shakespeare and his contemporaries…” I believe that the new title is catchier and will attract larger readership.

**Response**
Good suggestion, we have made the change.

2. “In this paper we aim to model speech length variation in these plays as comprehensively as possible” is not specific enough. Explain exactly how your work is different from others. Using mixed model?

**Response**
We have added several sentences at the end of the Introduction to detail the innovations in the article.

3. You use the mode as the central tendency measure but mixed model assumes that the central tendency measure is the mean. I see a contradiction.

   **Response**
   There is no contradiction, as explained above the mode is a summary measure of a distribution, in other words it is just a derived variable based on measurements. When we use another summary statistic, the mean, to summarise a general tendency or pattern for the mode from different plays, it is no different to using the mean to summarise a range of values based on any variable. The question we are answering is something like this. The process of making plays produces a range of speech lengths and when summarised by modes is not the same for each play. So how do we summarise the distribution of modes across many different plays. The mean is a sensible and natural option, but others could have been chosen, even the mode! But statistical theory and methods for the mean are much more developed, available and understood so the mean was the preferred choice.
4. 136: “…with mixed modes..” Is it a typo? Why modes? Shouldn’t be models?

   **Response**
   Modes was correct, the idea we were trying to explain was that between the early and final period there was a transition period in which both kinds of plays were being produced, those with high modes and those with low.
   The word mixed was been expanded now reading
   “1597-1602 plays (with a mixture of high and low modes)”

Approach

- A rebuttal letter that responds to each point raised by the academic editor and reviewer(s). You should upload this letter as a separate file labeled 'Response to Reviewers'.
- A marked-up copy of your manuscript that highlights changes made to the original version. You should upload this as a separate file labeled 'Revised Manuscript with Track Changes'.
- An unmarked version of your revised paper without tracked changes. You should upload this as a separate file labeled 'Manuscript'.

Thank you for submitting your manuscript to PLOS ONE. After careful consideration, we feel that it has merit but does not fully meet PLOS ONE’s publication criteria as it currently stands. Therefore, we invite you to submit a revised version of the manuscript that addresses the points raised during the review process.

I found the work interesting and worth of publication. However, before sending this paper out to reviewers I read it myself and came to conclusion that some critical modifications and revisions are to be made before giving the paper consideration for publication.

If you would like to make changes to your financial disclosure, please include your updated statement in your cover letter. Guidelines for resubmitting your figure files are available below the reviewer comments at the end of this letter.

If applicable, we recommend that you deposit your laboratory protocols in protocols.io to enhance the reproducibility of your results. Protocols.io assigns your protocol its own identifier (DOI) so that it can be cited independently in the future. For instructions see: [https://journals.plos.org/plosone/s/submission-guidelines#loc-laboratory-protocols](https://nam12.safelinks.protection.outlook.com/?url=https%3A%2F%2Fjournals.plos.org%2Fplosone%2Fs%2Fsubmission-guidelines%23loc-laboratory-protocols&data=05%7C01%7C%7Ca19c5a766fc34aa6e80808da61363d9f%7C84df9e7fe9f640afb435aaaaaaaaaaaa%7C1%7C0%7C637929183331180980%7CUnknown%7CTWFpbGZsb3d8eyJWIjoiMC4wLjAwMDAiLCJQIjoiV2luMzIiLCJBTiI6Ik1haWwiLCJXVCI6Mn0%3D%7C3000%7C%7C%7C&sdata=EcaBBkMvFw8LruhsYG9vLreBhN8OeLauxqe%2FuWlVEJk%3D&reserved=0). Additionally, PLOS ONE offers an option for publishing peer-reviewed Lab Protocol articles, which describe protocols hosted on protocols.io. Read more information on sharing protocols at [https://plos.org/protocols?utm_medium=editorial-email&utm_source=authorletters&utm_campaign=protocols](https://nam12.safelinks.protection.outlook.com/?url=https%3A%2F%2Fplos.org%2Fprotocols%3Futm_medium%3Deditorial-email%26utm_source%3Dauthorletters%26utm_campaign%3Dprotocols&data=05%7C01%7C%7Ca19c5a766fc34aa6e80808da61363d9f%7C84df9e7fe9f640afb435aaaaaaaaaaaa%7C1%7C0%7C637929183331180980%7CUnknown%7CTWFpbGZsb3d8eyJWIjoiMC4wLjAwMDAiLCJQIjoiV2luMzIiLCJBTiI6Ik1haWwiLCJXVCI6Mn0%3D%7C3000%7C%7C%7C&sdata=1dAXlfGrPDubR36%2BbkSTxHElfvVUSTkCGiD9ca7pzqQ%3D&reserved=0).

We look forward to receiving your revised manuscript.

Kind regards,

Eugene Demidenko, Ph.D.
Academic Editor
PLOS ONE

Journal requirements:

When submitting your revision, we need you to address these additional requirements.

1. Please ensure that your manuscript meets PLOS ONE's style requirements, including those for file naming. The PLOS ONE style templates can be found at 
[https://journals.plos.org/plosone/s/file?id=wjVg/PLOSOne_formatting_sample_main_body.pdf](https://nam12.safelinks.protection.outlook.com/?url=https%3A%2F%2Fjournals.plos.org%2Fplosone%2Fs%2Ffile%3Fid%3DwjVg%2FPLOSOne_formatting_sample_main_body.pdf&data=05%7C01%7C%7Ca19c5a766fc34aa6e80808da61363d9f%7C84df9e7fe9f640afb435aaaaaaaaaaaa%7C1%7C0%7C637929183331180980%7CUnknown%7CTWFpbGZsb3d8eyJWIjoiMC4wLjAwMDAiLCJQIjoiV2luMzIiLCJBTiI6Ik1haWwiLCJXVCI6Mn0%3D%7C3000%7C%7C%7C&sdata=5pGqsk3hxdoIourjMtuoyjhKghbSpAzEA8vn7XroX7I%3D&reserved=0) and 
[https://journals.plos.org/plosone/s/file?id=ba62/PLOSOne_formatting_sample_title_authors_affiliations.pdf](https://nam12.safelinks.protection.outlook.com/?url=https%3A%2F%2Fjournals.plos.org%2Fplosone%2Fs%2Ffile%3Fid%3Dba62%2FPLOSOne_formatting_sample_title_authors_affiliations.pdf&data=05%7C01%7C%7Ca19c5a766fc34aa6e80808da61363d9f%7C84df9e7fe9f640afb435aaaaaaaaaaaa%7C1%7C0%7C637929183331180980%7CUnknown%7CTWFpbGZsb3d8eyJWIjoiMC4wLjAwMDAiLCJQIjoiV2luMzIiLCJBTiI6Ik1haWwiLCJXVCI6Mn0%3D%7C3000%7C%7C%7C&sdata=Dt2W72BItM6Dg3uLSstbeikzcFC0%2BpI5OVabKohV6RM%3D&reserved=0)

2. Please ensure that you refer to Figure 7 in your text as, if accepted, production will need this reference to link the reader to the figure.

**Response**

Figure 7 is now referred to in the text.

3. We note you have included a table to which you do not refer in the text of your manuscript. Please ensure that you refer to Table 4 in your text; if accepted, production will need this reference to link the reader to the Table.

**Response**

Table 4 is now referred to in the text.

5.Please include captions for your Supporting Information files at the end of your manuscript, and update any in-text citations to match accordingly. Please see our Supporting Information guidelines for more information: [http://journals.plos.org/plosone/s/supporting-information](https://nam12.safelinks.protection.outlook.com/?url=http%3A%2F%2Fjournals.plos.org%2Fplosone%2Fs%2Fsupporting-information&data=05%7C01%7C%7Ca19c5a766fc34aa6e80808da61363d9f%7C84df9e7fe9f640afb435aaaaaaaaaaaa%7C1%7C0%7C637929183331180980%7CUnknown%7CTWFpbGZsb3d8eyJWIjoiMC4wLjAwMDAiLCJQIjoiV2luMzIiLCJBTiI6Ik1haWwiLCJXVCI6Mn0%3D%7C3000%7C%7C%7C&sdata=bAzkDWRWiyQQAs31%2FG53g7AJ%2FZK7G%2FFSA2TrPn1O5SU%3D&reserved=0)

**Response**

These are included in Section 6 at the end of the manuscript.

5. Please upload a copy of all your Supporting Information Figure which you refer to in your text.

**Response**

This appears to be necessary only in the case of S1 Fig 11 as all the other Supporting Information figures which are referred to in the text are identical to one of the main text figures included and the equivalent main-text figure number is linked to the S1 Fig number in each case. We will upload S1 Fig 11.
