# Supplementary material for: Changes in the length of speeches in the plays of William Shakespeare and his contemporaries: A mixed models approach
Source: PLoS One. 2023 Apr 21;18(4):e0282716. doi: 10.1371/journal.pone.0282716 (PMC10121026; doi:10.1371/journal.pone.0282716)
Supplement: Attachment — Submitted filename: Comments for the authors.docx [file pone.0282716.s010.docx]

Comments

This manuscript demonstrates an interesting attempt of applying statistical methods to analyzing the development trends in literary studies. Informative research findings include a shift towards shorter speeches used in plays and the possible influence of writing fashion. My suggestions focus on the statistical analysis section of this study:

1. “The length of words in speeches”, as was mentioned on Page 1, is the outcome variable/independent variable of the study. The authors have endeavored to provide a clear definition of “speech” on Page 3. Personally I think the readers would benefit from an example from the play scripts. “Speech” is often measured as the number of syllables between identifiable pauses in applied linguistics, while the number of words is the major index for evaluating speech length. In the literature review section, 2007 MacDonald P Jackson adopted a different system in calculating speech length—3-6 words/3-10 words were categorized as a speech. The authors have also made the argument of why syllabification is not used in this study on Page 8. It would help the readers, however, to dissolve possible doubts and keep the measurement of speech consistent if explanations appear at an earlier place in this manuscript.
2. I appreciate the detailed explanation of statistical methods from Page 11 to Page 14. However, this section could be reduced to some extent, as readers who have background knowledge in quantitative analysis usually understand how linear mixed models work. The selection of variables, or the identification of key predictors for speech length, requires a large amount of justification. This is in connection with the literature review section. I would recommend that the researchers dedicate more efforts to investigating previous studies discussing possible predictors of speech length, such as authorship, genre, etc. It is highly possible that not a lot of quantitative studies could be found, as statistical analysis might not have been the main research paradigm in literary studies. However, researchers’ discussion of possible factors would still help answer the major question of this study: which factors need to be included (or excluded) from the linear mixed model and why?
3. As for the discussion and implication section, my suggestions originate more from an applied linguistic approach. In addition to identifying significant predictors, the function of linear mixed models also lies in sorting out the most powerful factors rendering the final outcome variable. In the discussion section, instead of starting with an audience perspective, the authors could briefly report the coefficients in the model and discuss significant predictors and most influential predictors, which might be corroborated by the scripts used as examples.

It is exciting to see quantitative methods are also playing a role in literary research, and interdisciplinary studies were conducted in-depth with the help of statistics. The innovative methodology deserves a more prominent place in the implication part, which hopefully would be assist researchers with similar needs in their academic inquires.
