# Supplementary material for: Changes in the length of speeches in the plays of William Shakespeare and his contemporaries: A mixed models approach
Source: PLoS One. 2023 Apr 21;18(4):e0282716. doi: 10.1371/journal.pone.0282716 (PMC10121026; doi:10.1371/journal.pone.0282716)
Supplement: Attachment — Submitted filename: PLOS ONE reports with to do list v4.docx [file pone.0282716.s011.docx]

Reviewer # 1: Comments

This manuscript demonstrates an interesting attempt of applying statistical methods to analyzing the development trends in literary studies. Informative research findings include a shift towards shorter speeches used in plays and the possible influence of writing fashion. My suggestions focus on the statistical analysis section of this study:

1. “The length of words in speeches”, as was mentioned on Page 1, is the outcome variable/independent variable of the study. The authors have endeavored to provide a clear definition of “speech” on Page 3. Personally I think the readers would benefit from an example from the play scripts. “Speech” is often measured as the number of syllables between identifiable pauses in applied linguistics, while the number of words is the major index for evaluating speech length. In the literature review section, 2007 MacDonald P Jackson adopted a different system in calculating speech length—3-6 words/3-10 words were categorized as a speech. The authors have also made the argument of why syllabification is not used in this study on Page 8. It would help the readers, however, to dissolve possible doubts and keep the measurement of speech consistent if explanations appear at an earlier place in this manuscript.

**>We have added some detail on our definition of a speech and given an example, as suggested by >the reviewer, at ll. 55-65. We now make it clear that Jackson 2007 was selecting from among >available speeches, rather than adopting a different system to calculate speech length, as the >reviewer wrongly understood, at ll. 131-3. As the reviewer recommended, we have inserted a >brief preliminary defence of the choice to count words rather than syllables, at ll. 87-92.**

2. I appreciate the detailed explanation of statistical methods from Page 11 to Page 14. However, this section could be reduced to some extent, as readers who have background knowledge in quantitative analysis usually understand how linear mixed models work.

**>We have interpreted this to mean mostly the mixed model theory related description rather than >the methods used for the model fitting and have trimmed this section. 43 lines are now reduced >to 24 and 628 words reduced to 342. See ll. 369-418.**

The selection of variables, or the identification of key predictors for speech length, requires a large amount of justification. This is in connection with the literature review section. I would recommend that the researchers dedicate more efforts to investigating previous studies discussing possible predictors of speech length, such as authorship, genre, etc. It is highly possible that not a lot of quantitative studies could be found, as statistical analysis might not have been the main research paradigm in literary studies. However, researchers’ discussion of possible factors would still help answer the major question of this study: which factors need to be included (or excluded) from the linear mixed model and why?

**>We have revised Section 2.2.2 (ll. 224-287), now describing more fully the motivation for the >choice of each variable.**

3. As for the discussion and implication section, my suggestions originate more from an applied linguistic approach. In addition to identifying significant predictors, the function of linear mixed models also lies in sorting out the most powerful factors rendering the final outcome variable. In the discussion section, instead of starting with an audience perspective, the authors could briefly report the coefficients in the model and discuss significant predictors and most influential predictors, which might be corroborated by the scripts used as examples.

**>We have added a new section, 4.1, “Importance of effects”, of about a page, to respond to this >suggestion, and renumbered the following sections to match.**

It is exciting to see quantitative methods are also playing a role in literary research, and interdisciplinary studies were conducted in-depth with the help of statistics. The innovative methodology deserves a more prominent place in the implication part, which hopefully would be assist researchers with similar needs in their academic inquires.

**>The methodology is now cited as one of the important implications of the study at the beginning >of Section 4.4, ll. 991-1001.**

Reviewer # 2: Manuscript Number: PONE-D-22-17959R1
Manuscript Title: Changes in the length of speeches in the plays of William Shakespeare and his contemporaries: a mixed models approach

The article does a good job of establishing its findings in a succinct and easy-to-understand manner. However, there were a few issues that need to be revised. There are as follows:

Major issues:
While the overall quality of the article is good and it is scientifically sound, quite a few claims made in the article are unsubstantiated.
For instance:- the sentence ‘The change in speech lengths is part of a wider collective drift in the plays towards liveliness and verisimilitude and is evidence of a hitherto hidden constraint on the playwrights.’ might be accurate, it would do well to establish this with a few instances of this so-called ‘hidden constraint’ or ‘hidden patterns’ as mentioned in section 1.

**>We have clarified with more explanation at ll. 35-6.**

Similarly, the phrase ‘publishers who applied other equally well-established typographical conventions to mark off the speeches when printing plays from manuscripts.’ In section 1 would also do well by adding a few examples of these ‘typographical conventions’ to substantiate the claim of the sentence. (i.e. everyone knows there are certain ‘typographical conventions to mark off speeches’, but in a scientific article, these do need to be explicitly mentioned, even if in passing.)

**>Examples of typographical and manuscript conventions to mark off speeches are now given at ll. >72-75.**

The sentence ‘Comparatively long speeches, such as soliloquies and speeches reporting off-stage events, declaring sentiments, or attempting to persuade, have attracted the most commentary to date, so we might assume this sort of length is dominant.’ In section 1 is another example of a sentence making a sweeping remark without substantiating it. (While it be accurate that longer speeches attract more commentary, in a scientific article you need to back that up with facts and proof, either referencing from where you derive this information, or making this analysis yourself from the data you have.)

**>We have modified the text at ll. 78-79 to make the remark less sweeping and when returning to >the same issue in Section 4.4 have re-framed the context and added two references to >substantiate the claims (ll. 1003-10).**

There are quite a few sentences in the article that share this issue, and they stick out like a sore thumb in an otherwise well-writ and researched article. I would suggest that the authors take some time to go through the article to either substantiate (with valid references), or get rid of, unsubstantiated claims.

**>We have added references at strategic points. There are now 42 references compared to 29 in >the earlier version.**

Minor issues:
The article is afflicted with several grammatical and spelling issues, which is unbecoming of a scientific article, specially one that deals with William Shakespeare’s plays. It would be better to go through the article closely and remove any syntactic inaccuracies. A few examples of these are :
in section 1.2, ‘ Ilsemann observed that in these plays the commonest speech length …’
shouldn’t it be ‘most common’ instead of ‘commonest’, as it is much widely used and also avoids ambiguity? In a similar fashion, in the same section, the sentence ‘Lines shared between speakers were rare in Shakespeare’s earliest plays, making up two per cent or so of the total, but became more common in his middle period, and commoner still at the end, with the last plays.’ Makes use of the word ‘commoner’, rather than ‘even more common’. The word ‘commoner’, specially in an article pertaining to Shakespearean plays, could easily be misinterpreted as ‘a common person, as distinguished from one with rank, status, etc.’ and introduce ambiguity in a otherwise lucid and well-articulated article.
In the abstract, the sentence ‘We confirm that that the mode …’ falls prey to one of the more common errors that occur while typing or writing anything in English. The dreaded ‘that that’ in the abstract of an scientific article is disappointing.

I would suggest the authors to comb through the article and rid it of these minor issues before the article is ready for publication.

**>We have corrected all the specific errors noted and corrected stylistic errors throughout after a >further proof-reading process.**
